# Air Pollution, housing and respiratory tract Infections in Children: NatIonal birth Cohort study (PICNIC): study protocol

**DOI:** 10.1136/bmjopen-2020-048038

**Published:** 2021-05-03

**Authors:** Graziella Favarato, Tom Clemens, Steven Cunningham, Chris Dibben, Alison Macfarlane, Ai Milojevic, Jonathon Taylor, Linda Petronella Martina Maria Wijlaars, Rachael Wood, Pia Hardelid

**Affiliations:** 1 Population, Policy and Practice Research and Teaching Department, University College London Institute of Child Health, London, UK; 2 School of Geosciences, The University of Edinburgh, Edinburgh, UK; 3 Centre for Inflammation Research, University of Edinburgh, Edinburgh, UK; 4 Department of Midwifery, City University, London, UK; 5 Department of Social and Environmental Health Research, London School of Hygiene & Tropical Medicine, London, UK; 6 Faculty of Built Environment, Tampere University, Tampere, Finland; 7 Clinical and Public Health Intelligence Team, Public Health Scotland, Edinburgh, UK; 8 Centre for Clinical Brain Sciences, University of Edinburgh, Edinburgh, UK

**Keywords:** epidemiology, public health, paediatrics, respiratory infections

## Abstract

**Introduction:**

Respiratory tract infections (RTIs) are the most common reason for hospital admission among children <5 years in the UK. The relative contribution of ambient air pollution exposure and adverse housing conditions to RTI admissions in young children is unclear and has not been assessed in a UK context.

**Methods and analysis:**

The aim of the PICNIC study (Air Pollution, housing and respiratory tract Infections in Children: NatIonal birth Cohort Study) is to quantify the extent to which in-utero, infant and childhood exposures to ambient air pollution and adverse housing conditions are associated with risk of RTI admissions in children <5 years old. We will use national administrative data birth cohorts, including data from all children born in England in 2005–2014 and in Scotland in 1997–2020, created via linkage between civil registration, maternity and hospital admission data sets. We will further enhance these cohorts via linkage to census data on housing conditions and socioeconomic position and small area-level data on ambient air pollution and building characteristics. We will use time-to-event analyses to examine the association between air pollution, housing characteristics and the risk of RTI admissions in children, calculate population attributable fractions for ambient air pollution and housing characteristics, and use causal mediation analyses to explore the mechanisms through which housing and air pollution influence the risk of infant RTI admission.

**Ethics, expected impact and dissemination:**

To date, we have obtained approval from six ethics and information governance committees in England and two in Scotland. Our results will inform parents, national and local governments, the National Health Service and voluntary sector organisations of the relative contribution of adverse housing conditions and air pollution to RTI admissions in young children. We will publish our results in open-access journals and present our results to the public via parent groups and social media and on the PICNIC website. Code and metadata will be published on GitHub.

**Strengths and limitations of this study**

- The PICNIC study will use national, administrative data birth cohorts from England and Scotland, linked to small area-level data on environmental exposures and census data on socioeconomic position, to examine the association between ambient air pollution and adverse housing exposures and respiratory tract infection admissions in children less than 5 years old.
- The national birth cohorts will include all children born in the two countries during specified time periods, thus minimising selection bias and allowing analyses of even relatively uncommon environmental exposures and infection outcomes.
- Data on air pollution and building characteristics will be linked to maternal and child postcode histories during pregnancy and early life, thus creating longitudinal environmental exposure data at a national scale.
- PICNIC will include an examination of the population-based risk factors for SARS-CoV-2 infection in Scottish children.
- A key weakness is that only infections requiring hospital admission will be the primary outcome, thus respiratory infections not requiring healthcare contact will not be considered.

## Introduction

Upper and lower respiratory tract infections (RTIs), including croup, bronchiolitis and pneumonia, are the most common reason for hospital admission in children aged less than 5 years old in the UK, with 170 000 admissions in England alone in 2017.^1^ RTI admission rates peak in winter months, contributing to the ‘winter crisis’ in the National Health Service (NHS).^2^ Severe RTI symptoms in infancy and early childhood have been linked to adverse respiratory health outcomes in later childhood, including asthma.^3^ We have previously shown that 79% of annual admissions for RTIs in infants <1 year old can be attributed to respiratory syncytial virus (RSV) and other viral infections for which no vaccines are currently available.^4^ Thus, alternative strategies to vaccination are required to prevent RTIs and relieve the burden on children, parents and the NHS.

**Figure 1:**
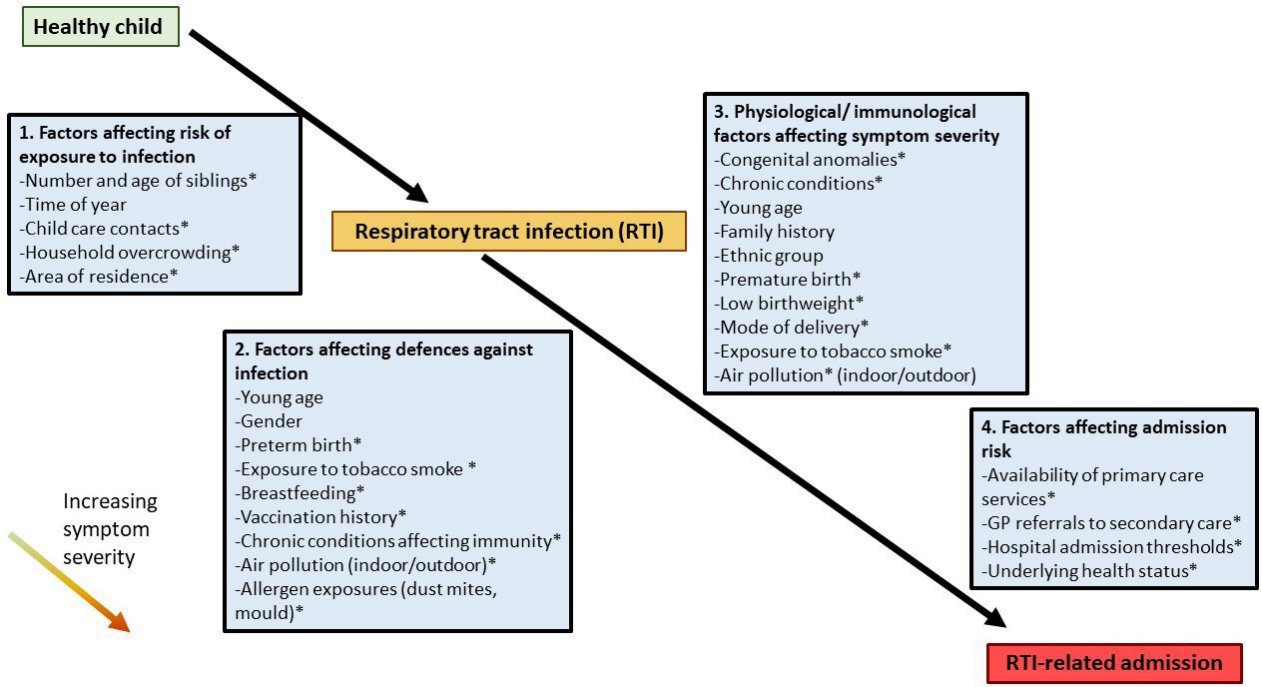
Factors which increase the risk of admissions for respiratory tract infection in children. Adapted from Heikkinen and Chonmaitree.^7^ *Proximal risk factors for which low socioeconomic position is an upstream correlate. GP, general practitioner.

Low socioeconomic position (SEP) is strongly associated with the risk of RTI admissions in children, even when accounting for tobacco smoke exposure.^5 6^ There are a number of well-established proximal risk factors for RTI admissions through which low SEP may act to increase the risk of infection exposure, severe symptoms and hospital admission (figure 1, framework adapted from Heikkinen and Chonmaitree).^7^

Of these risk factors, exposures to ambient air pollution and adverse housing conditions (including overcrowding, indoor air pollution and damp/mould) are of particular policy and research interest in the UK. The UK has been breaching European Union targets for nitrogen dioxide (NO_2_) levels for over 10 years,^8^ and 18% of homes in England do not meet the government’s Decent Homes Standard.^9^ A number of studies have shown that exposure to ambient air pollution (particularly fine particulate matter (PM_2.5_) and NO_2_) during pregnancy and early life^10 11^ and living in overcrowded^12^ or damp/mouldy housing^13^ increase the risk of RTIs in children. However, their joint contribution to RTI admissions in children has not been assessed in a UK context.

### Aims and objectives

The overall aim of the PICNIC study (Air Pollution, housing and respiratory tract Infections in Children: NatIonal birth Cohort) is to determine the relative contribution of ambient air pollution and adverse housing conditions to the rate of RTI-related hospital admissions in children aged less than 5 years old. Since 44% of RTI admissions in children under 5 years old occur during infancy (the first year of life),^4^ we will first focus on infant admissions, then extend our analyses until 4 years of age inclusive. We will use administrative data birth cohorts covering all children born in England and Scotland, enhanced via linkage to data from the corresponding censuses and small area-level data on ambient air pollution and building characteristics.

Our specific objectives are to:

1. Estimate the association between long-term exposure to ambient air pollution during pregnancy and infancy and the rate of RTI admissions in infants.
2. Derive variables indicating adverse housing conditions (eg, overcrowding, air pollution indoors, damp/mould, ventilation) and estimate the association between housing exposures during pregnancy and infancy and infant RTI admissions.
3. Based on the outcomes of analyses for objectives 1 and 2, estimate the relative contribution of ambient air pollution and adverse housing conditions to infant RTI admissions.
4. Estimate the relative contribution of ambient air pollution and adverse housing conditions exposure during pregnancy, infancy and early childhood to RTI admissions in children less than 5 years old.
5. Establish the linked administrative data birth cohorts as resources for maternal and child health research.

Since receipt of funding we have added two further objectives to the study:

1. Examine the association between environmental, clinical and socioeconomic risk factors and COVID-19 and other respiratory viruses in children and young people (this objective has been added as a response to the COVID-19 pandemic).
2. Estimate the relative contribution of ambient air pollution and adverse housing conditions to community dispensed medicines for children aged less than 5 years old.

Due to data availability, objectives 6 and 7 will be addressed using data from Scotland only.

## Proposed methods

### Study design, study population and follow-up

PICNIC is a population-based birth cohort study. The PICNIC study population includes two national birth cohorts comprising all children born in England between 2005 and 2014 inclusive and in Scotland between 1997 and 2020 inclusive. Children will be followed from the discharge date of their postnatal admission until their date of death, migration out of Scotland or England (defined using the methods described in Hardelid *et al*^6^ for Scotland and Lewis *et al*^2^ for England), or their fifth birthday, whichever occurs first, via hospital admission and death records. The Scottish birth cohort will also include longitudinal data on community dispensed drugs, microbiology test results, vaccination and health visiting (see the following sections).

### Data sources

#### Birth cohorts from England and Scotland

**Figure 2:**
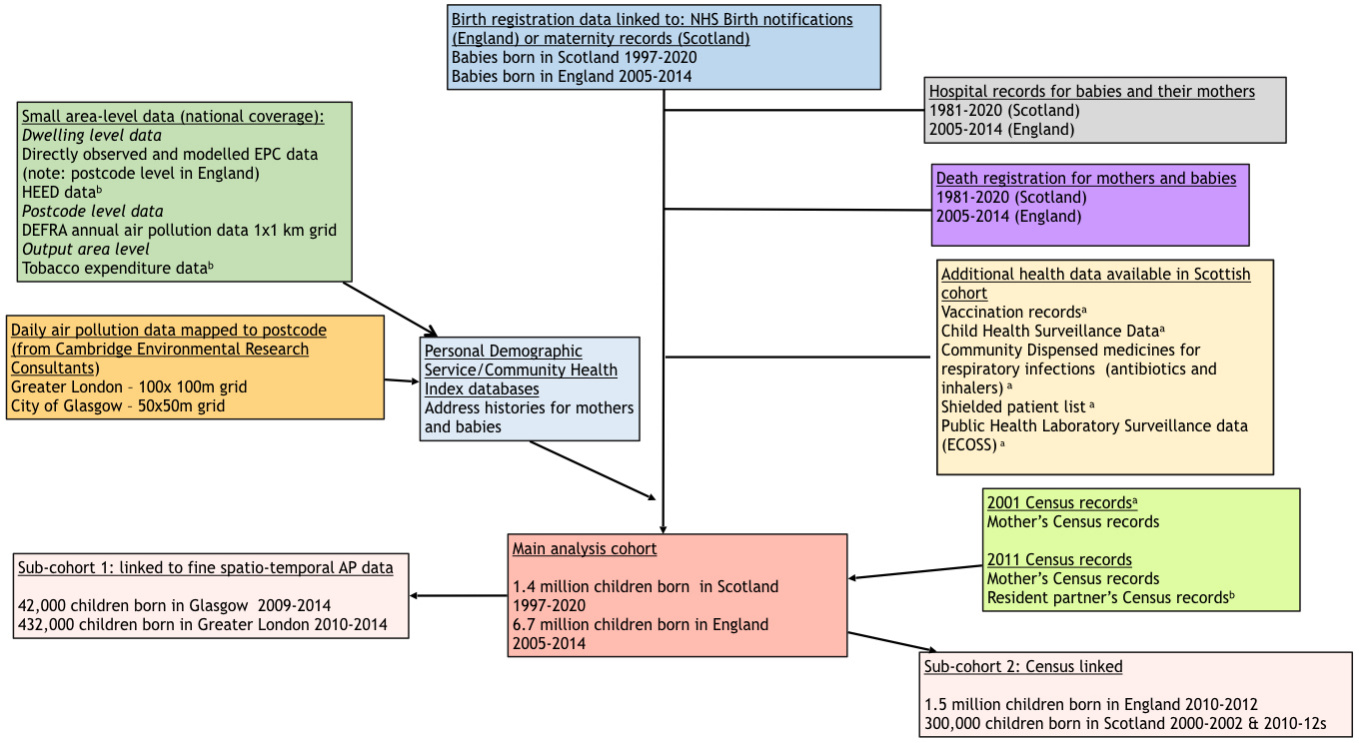
Flow chart of linked data sources in PICNIC study. ^a^Scotland only. ^b^England only. AP, air pollution; DEFRA, Department for Environment, Food and Rural Affairs; ECOSS, Electronic Communication of Surveillance in Scotland; EPC, Energy Performance Certificates; HEED, Home Energy Efficiency Database; NHS, National Health Service.

The *English birth cohort* covers all 6.7 million births in England between 2005 and 2014. It is based on linkage between Office for National Statistics (ONS) live birth and stillbirth, and death registration, NHS birth notifications and longitudinal Hospital Episode Statistics (HES) records for mothers and babies (figure 2). The data linkage methods used to create the cohort were developed for a project led by City, University of London.^14^ The methods to clean and validate the cohort have been described elsewhere.^15–17^

For the *Scottish birth cohort*, the electronic Data Research and Innovation Service (eDRIS) will link data from Scottish birth registrations to maternity records (Scottish Morbidity Record, SMR-02), Scottish Birth Record (and its predecessor SMR-11), death registration and longitudinal hospital data (SMR-01) for the mother and the baby. Linkage of these five data sets will allow the derivation of similar risk factors to England, but importantly SMR-02 also includes individual-level data on maternal smoking during pregnancy. The sophisticated Scottish health data linkage infrastructure will also allow linkage to information on breast feeding and tobacco smoke exposure at child health first visit and at 6–8 weeks of age (from the Child Health Surveillance Programme-Preschool; CHSP-PS) and routine infant vaccinations from the Scottish Immunisation and Recall System. We have requested linked data from eDRIS on births from 1997 onwards (when the new SMR-02 record was introduced), resulting in a cohort of approximately 1.4 million births.

#### Additional linkage to health data in Scotland in response to COVID-19 crisis

In response to the COVID-19 pandemic, we will seek to extend the linkage of the Scottish cohort to a number of further data sets. In Scotland, the birth cohort will also be linked to public health laboratory testing data (via the Electronic Communication of Surveillance in Scotland^18^) and the list of COVID-19 shielded patients. In the UK, people were flagged as shielded if they were extremely vulnerable to COVID-19. These primarily included people who were substantially immunosuppressed, including those with cystic fibrosis or solid organ transplant recipients. These linkages will allow us to address objective 6.

Further, we will extend the cohort in Scotland to include community dispensing data for antibiotics (focusing particularly on amoxicillin, phenoxymethylpenicillin or erythromycin) or asthma inhalers (beta_2_ agonists and inhaled corticosteroids) via linkage to the Prescribing Information System.^19^ Three-quarters of antibiotics prescribed to children in primary care in the UK are for RTIs.^20^ Therefore, although not all dispensing of amoxicillin, phenoxymethylpenicillin or erythromycin are for RTIs, dispensing of these antibiotics will serve as a useful proxy. These further linkages will allow us to address additional objective 7.

#### Longitudinal address records

Linkage of the birth cohort to the Personal Demographic Service (PDS; in England) and Community Health Index database (CHI; in Scotland) will allow us to assign longitudinal air pollution and housing exposures to the mothers and babies in the cohort. PDS and CHI represent the most complete record of address histories for all NHS patients in England^21^ and Scotland,^22^ respectively.

#### Air ambient pollution data

We will primarily use Department for Environment, Food and Rural Affairs (DEFRA) open access data for annual background concentrations of eight major air pollutants.^23^ These air pollution concentrations have been derived from atmospheric chemistry transport models and are mapped to a 1×1 km grid across the UK for the period 2001–2019. These data have been widely used for studying the health effects of long-term exposure to air pollution in the UK.^24 25^

To take into account smaller spatial variation in exposure from traffic-derived air pollution, we will use data from OpenStreetMap combined with data on traffic density and modelled data provided by the Cambridge Environmental Research Consultants (CERC; http://www.cerc.co.uk). CERC will provide daily resolution data for PM_2.5_, PM_10_, NO_2_ and ozone (O_3_)for Greater London (modelled at a 100×100 m grid) and the City of Glasgow (modelled at a 50×50 m grid) for the years 2010–2014 and 2009–2014, respectively.

We will use road network data openly available from OpenStreetMap^26^ to calculate distance from each mother/child postcode centroid to major roads (motorways and A roads). Distance estimates will be combined with open, geocoded traffic intensity data from the Department for Transport^27^ on annual daily flows for different vehicle types between junctions on major roads.

#### Housing data sources

##### Census

The decennial censuses for the constituent nations of the UK contain rich data on housing (eg, tenure, accommodation type, central heating type, number of rooms and occupants) as well as data on information on self-reported health and SEP (including education, car ownership and employment). A subset of births in the English cohort between 2010 and 2012 have been linked by ONS to 2011 Census data provided by the mother, and the mother’s resident partner (if any) on the 2011 Census date (27 March). Mothers in the Scottish cohort who gave birth between 2000 and 2002 will be linked to their 2001 Census records (which took place on 29 April), and mothers giving birth between 2010 and 2012 will be linked to their 2011 Census records.

##### Energy performance certificates

*Energy performance certificates (EPCs)* contain energy efficiency data for all buildings in England, Wales and Scotland constructed, let or sold since 2007,^28^ currently approximately 11.5 million unique dwellings. The EPC databases contain directly observed information on building characteristics at dwelling level, including energy efficiency rating, insulation levels and total floor area. EPC data for England are openly available online; EPC data for Scotland will be provided by the Energy Savings Trust (https://energysavingtrust.org.uk/scotland) on behalf of the Scottish Government. Apart from directly observed variables from EPC, we will use building physics models^29^ with inputs from the EPC database to predict indoor environmental conditions at postcode level nationally for England and Scotland, including indicators of mould/damp and indoor air pollution exposures. The *Home Energy Efficiency Database (HEED*) is also held by the Energy Savings Trust Scotland and contains Scotland-wide information on energy efficiency measures installed with government grants since 2009, including double glazing and roof insulation.

#### Tobacco expenditure data

Unlike in the Scottish cohort, individual-level data on tobacco smoke exposure during pregnancy or early life are not available in the English cohort. We will instead use modelled data on tobacco expenditure at the output area level (on average 125 households), from CACI (https://www.caci.co.uk). These data have been used previously in studies on the association of environmental hazards and children’s health outcomes in England to adjust for tobacco smoke exposure.^30^

### Data linkage

The suggested data flows and linkages for the English and Scottish cohorts are summarised in online supplemental appendix figures 1–3.

10.1136/bmjopen-2020-048038.supp1 Supplementary data

10.1136/bmjopen-2020-048038.supp2 Supplementary data

10.1136/bmjopen-2020-048038.supp3 Supplementary data

For the *English cohort* (online supplemental appendix figures 1 and 2), the linkage to set up the birth cohort has been described previously.^14 16^ ONS has linked mothers’ information recorded on ONS birth records to the 2011 Census using a method based on names, postcodes and dates of birth.^31^ Mothers’ census records have been linked to those of their partner using the census household matrix. NHS Digital will link the maternal and child identifiers from the birth cohort to the PDS using mother and child NHS numbers, dates of birth and postcode at delivery/birth. Air pollution exposures and EPC data will be aggregated at postcode level for linkage to the birth cohort via the longitudinal postcode records in PDS.

For the *Scottish cohort* (online supplemental appendix figure 3), eDRIS will use well-established deterministic and probabilistic methods to link the health data sets for PICNIC. National Records of Scotland will use a similar method to ONS to link the maternal information to 2001 and 2011 Census records using names, dates of birth and postcodes at delivery. As part of the COVID-19 pandemic response, eDRIS has linked the CHI database to Unique Property Reference Numbers (UPRNs), which uniquely identify individual dwellings. In Scotland, HEED and EPC records will therefore be linked to the birth cohort via CHI using UPRNs, rather than postcodes. Ambient air pollution exposures will be linked to the mother and baby address histories in CHI using postcodes.

#### Outcomes of interest

Our primary outcome will be RTI-related hospital admissions. We will identify admissions for upper RTIs (including croup, tonsillitis), lower RTIs (eg, pneumonia, bronchiolitis, viral wheeze) and procedures for chronic RTI symptoms (tonsillectomies and myringotomies) in the longitudinal hospital admission data available in the HES for the English birth cohort and SMR-01 for the Scottish birth cohort. Our secondary outcomes to be investigated using the Scottish cohort only are (1) community dispensed antibiotics and (2) asthma medicines, (3) positive test results for SARS-CoV-2, and (4) positive test results for other respiratory viruses, including influenza and RSV.

We will use gastrointestinal infections (GIs) as a negative control outcome for objective 1, since we hypothesise that GIs are not associated with air pollution exposure.^32^

### Exposure variables

#### Outdoor air pollution

We will derive air pollution exposures for PM_10_, PM_2.5_, NO_2_, nitrogen oxides (NO_x_), O_3_, sulphur dioxide (SO_2_), benzene and carbon monoxide (CO) using the DEFRA data mapped to the mother’s postcode during pregnancy and the child’s postcode during the first 5 years of life. The detailed CERC data for London and Glasgow will also be mapped to mother/child postcodes and used to calculate and explore the impact of trimester-specific exposures. We will treat the air pollution variables as continuous exposures in the analyses. We will use data on distance to road and traffic density to produce a ‘distance to high traffic flow’ variable to be included in the analyses.

#### Housing conditions

From census data, we will derive indicators of housing attributes, including number of people per bedroom (a measure of overcrowding), accommodation type (also available from EPC data) and tenure. From the building characteristics data, we will extract a number of variables, including double glazing, energy efficiency and connection to mains gas, and derive the infiltration rate (ie, an estimate of how draughty the dwelling is). From the modelled EPC data, we will use estimated indoor/outdoor air pollution ratio^33^ and probability of damp or mould. These exposures will be available at dwelling level in Scotland and at postcode level in England.

#### Additional variables

A number of variables that may confound or modify the association between air pollution, housing conditions and the risk of RTIs in children will be available from the linked data sets in PICNIC (see table 1).

**Table 1:** Potential confounding or effect-modifying variables available in the linked birth cohorts

Birth registration	Birth registrations linked to notifications (England)/maternity records (Scotland)	Longitudinal hospital admission data	Census 2001* and 2011†	Additional variables
Month of birth. Sex of baby. Parents’ occupation. Parents’ countries of birth. Birth weight. Marital status. Type of registration (joint/sole registration). Index of Multiple Deprivation^41^/Scottish Index of Multiple Deprivation^42^ (small area level (~1500 people) indicators of deprivation derived from residential postcode).	Gestational age. Recorded ethnic group of baby. Maternal smoking status.* * Apgar score at 5 min.* Parity.*	Maternal chronic conditions. Baby’s chronic conditions. Baby’s congenital anomalies. Length of stay of birth admission.	Employment status. Education level. Length of stay in the UK (if born abroad). Knowledge of English. Self-reported health. Disability. Car ownership.	Tobacco expenditure data (at output area level).‡ Infant vaccines received (from SIRS).* Breast feeding at 6–8 weeks (from CHSP-PS).* Ethnic group of baby (from CHSP-PS).* Secondhand tobacco smoke exposure (from CHSP-PS).*

Note parity has been derived in England using birth registration and longitudinal HES records.^14^

*Scotland only.

†For mother and (in England only) mother’s partner at the census date (if a partner is present in the household).

‡England only.

CHSP-PS, Child Health Surveillance Programme-Preschool; HES, Hospital Episode Statistics; SIRS, Scottish Immunisation and Recall System.

### Statistical analyses

For objective 1, we will fit appropriate time-to-event regression models to estimate the association between ambient air pollution exposures during pregnancy and infancy and the rate of RTI hospital admissions. We will derive in-utero air pollution exposure levels from the annual air pollution data according to the number of weeks in each trimester that were spent at each address and include these trimester-specific exposures as baseline covariates in the model. To separate the effects of in-utero air pollution exposure from air pollution exposures during infancy, we will include infant air pollution exposure as a time-varying covariate in the models. We will first fit single-pollutant models, adjusted for all relevant confounders (identified by drawing causal diagrams of all factors assumed to be associated with RTI admission), then only include pollutants associated with the rate of RTI admission in multipollutant models. We will repeat the models for road distance/traffic density exposures. For London and Glasgow, we will derive variables indicating trimester-specific exposures to NO_2_, PM_2.5_ and O_3_ using the CERC data with finer temporal and spatial resolution, and refit the models to examine whether critical exposure windows exist during pregnancy or infancy. We will carry out sensitivity analyses by refitting the models using GIs as a negative control outcome to examine the extent of unmeasured confounding.^34^

To estimate the association between the rate of RTI admissions and housing (objective 2), we will primarily use data for the subset of children with linked maternal census data; however, longitudinal EPC and HEED data will be available for the other children in the cohort. We will examine the distribution of housing and building variables among children to examine the degree to which they cluster and how building characteristics change over pregnancy and early childhood. We will use appropriate time-to-event regression models to model the association between exposure to adverse housing/building characteristics during pregnancy and infancy and the rate of RTI admissions in infancy. Depending on the outcome of our preliminary analyses, we will either enter each housing variable into the regression models separately, or first apply dimension reducing methods (eg, latent class models) to identify meaningful combinations of housing/building characteristics. If exposure to building characteristics changes substantially during childhood (due to residential moving), we will include these as time-varying covariates in the model. HEED data will be used to examine the potential impact of changes to housing conditions due to retrofitting and the subsequent impact on the risk of RTIs in children.

To achieve objective 3, we will fit appropriate time-to-event models examining the joint contribution of ambient air pollution and housing/building characteristics to RTI admissions in infancy, by including terms for the air pollution and housing variables found to be associated with RTI admissions in objectives 1 and 2, and air pollution:housing effect modification terms. We will calculate population attributable fractions for air pollution and housing characteristics^35^ and use causal mediation analyses^36^ to explore the mechanisms through which housing and air pollution influence the risk of infant RTI admission.

We will repeat analyses for objectives 1–3, extending the follow-up time until the child’s fifth birthday for objective 4. As for objectives 1–3, we will model time to first RTI event, but also examine the time to repeated events using methods for multivariate failure time data.^37^ RTI in infancy is associated with risk of repeated wheeze episodes in later childhood.^38^ Therefore we will examine whether exposure to ambient air pollution and/or adverse housing conditions increases the frequency of recurrent wheeze admissions (or salbutamol prescribing in the Scottish cohort) among children with an RTI admission in infancy.

The English and Scottish birth cohorts linked to census and small area-level data will be extremely rich longitudinal data sets that can be used to examine the long-term consequences of environmental exposures, housing and SEP during pregnancy and early childhood. To meet objective 5, we will develop clear governance procedures and documentation for researchers wishing to access the existing cohorts and/or link in other data sets. All our code and metadata will be published on our GitHub page (https://github.com/UCL-CHIG).

For objective 6, we will build on our previous work examining family and clinical risk factors for influenza^39^ and RSV,^6^ to calculate rates of SARS-CoV-2 virus positivity and associated hospital admissions in children according to clinical, family and environmental risk factors. For this objective, we will follow children from birth until 24 years of age. We will use appropriate time-to-event models to estimate the independent association between the risk factors and COVID-19 infection. We will repeat these analyses for other respiratory viruses, including influenza and RSV. As part of this objective, we will also explore the potential to link in additional data on SARS-CoV-2 infections among the child’s household contacts to further examine the epidemiology of SARS-CoV-2 among children.

For objective 7, we will repeat our analyses for objectives 3 and 4 using rates of community dispensed antibiotics and asthma inhalers as outcome variables.

### Patient and public involvement

We have carried out several consultations with parents during the development of this proposal:

1. Consulting parents in Bradford, via the Born in Bradford Study,^40^ on the control of respiratory infections in children (June 2016).
2. Meeting with the National Institute for National Research (NIHR) Great Ormond Street Biomedical Research Centre Parents’ Advisory Group (March 2017).
3. Consulting parents with a history of housing problems, Shelter Birmingham (June 2018).

Parent involvement has led to including housing exposures and examining the combined effect of adverse housing conditions and air pollution exposure. We will work with the Clean Air Parents Network and Shelter to set up meetings with parents who have been affected by poor housing or are concerned about the health effects of air pollution to guide analyses, interpret findings and support dissemination.

### Expected study size

1. For objective 1, we will use all available linked data: 6.7 million children in England (1 200 000 RTI admissions) and 1.4 million children in Scotland (~200 000 RTI admissions).
2. For objectives 2–4 (including building/housing characteristics), 1.5 million children have been linked to their mother’s 2011 Census records. In Scotland, around 300 000 children will be linked to their mother’s 2001 and 2011 Census records.
3. Around 384 000 children born in London in 2010–2014 and 42 000 in Glasgow in 2009–2014 will be linked to 100×100 m and 50×50 m grid daily air pollution exposure data, respectively.

## Ethics and dissemination

### Ethics and research governance

We have obtained or are in the process of obtaining ethical or information governance approvals from the following committees:

#### England

1. NHS London Queen Square Ethics Committee (full approval, reference: 18/LO/1514).
2. Confidentiality Advisory Group (full approval, reference: 18/CAG/0159).
3. Administrative Data Research Network (approval, reference PROJ-194; note committee is no longer operating).
4. ONS Research Accreditation Panel (full approval, reference 2019/020).
5. National Statistician’s Data Ethics Advisory Committee (full approval, reference: 18 (07)).
6. Independent Group Advising on Release of Data (NHS Digital; full approval, DARS-NIC-234656).

#### Scotland

1. Public Benefit and Privacy Committee-Health and Social Care (full approval, reference 1819-0049).
2. Public Benefit and Privacy Committee-Statistics (preliminary approval, reference 1819-0049).
3. University of Edinburgh School of Geosciences Ethics Committee (full approval, reference 2020-401).

### Data storage and access

All linked analyses data sets will be kept on secure servers at the ONS Secure Research Service and the Public Health Scotland National Safe Haven. All outputs (tables and figures) will be disclosure-checked before being exported from these secure servers. All researchers accessing data will require training in information governance and data security as specified by ONS and Public Health Scotland.

### Dissemination policy

Our research findings will be relevant to a broad audience of researchers from paediatric and pulmonary medicine, environment and climate research, and epidemiology and public health. We will therefore publish our findings in high-impact journals across these fields. Similarly, we will present our research at national and international conferences in a range of disciplines. Our findings will be summarised using animations with links to papers on the PICNIC study website (https://www.ucl.ac.uk/child-health/research/population-policy-and-practice-research-and-teaching-department/cenb-clinical-12), and we will work with the University College London (UCL) press office to disseminate our results to a broad audience. Cohort descriptions will be published in peer-reviewed journals.

## Supplementary Material

Reviewer comments

Author's manuscript
