# Supplementary figures and images for: Air Pollution, housing and respiratory tract Infections in Children: NatIonal birth Cohort study (PICNIC): study protocol
Source: BMJ Open. 2021 May 3;11(5):e048038. doi: 10.1136/bmjopen-2020-048038 (PMC8098990; doi:10.1136/bmjopen-2020-048038)

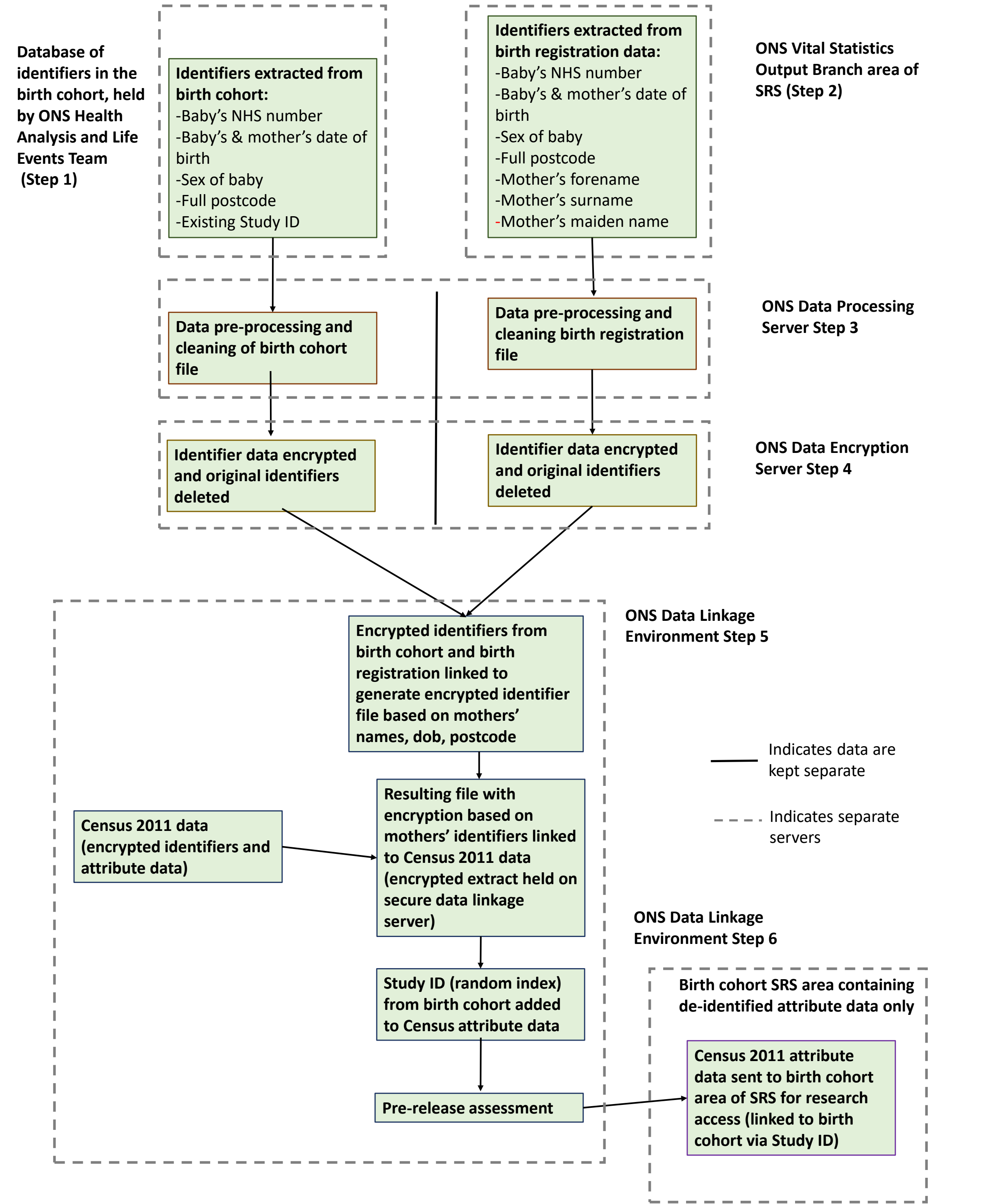

Supplement: Supplementary data [file bmjopen-2020-048038supp001.pdf]

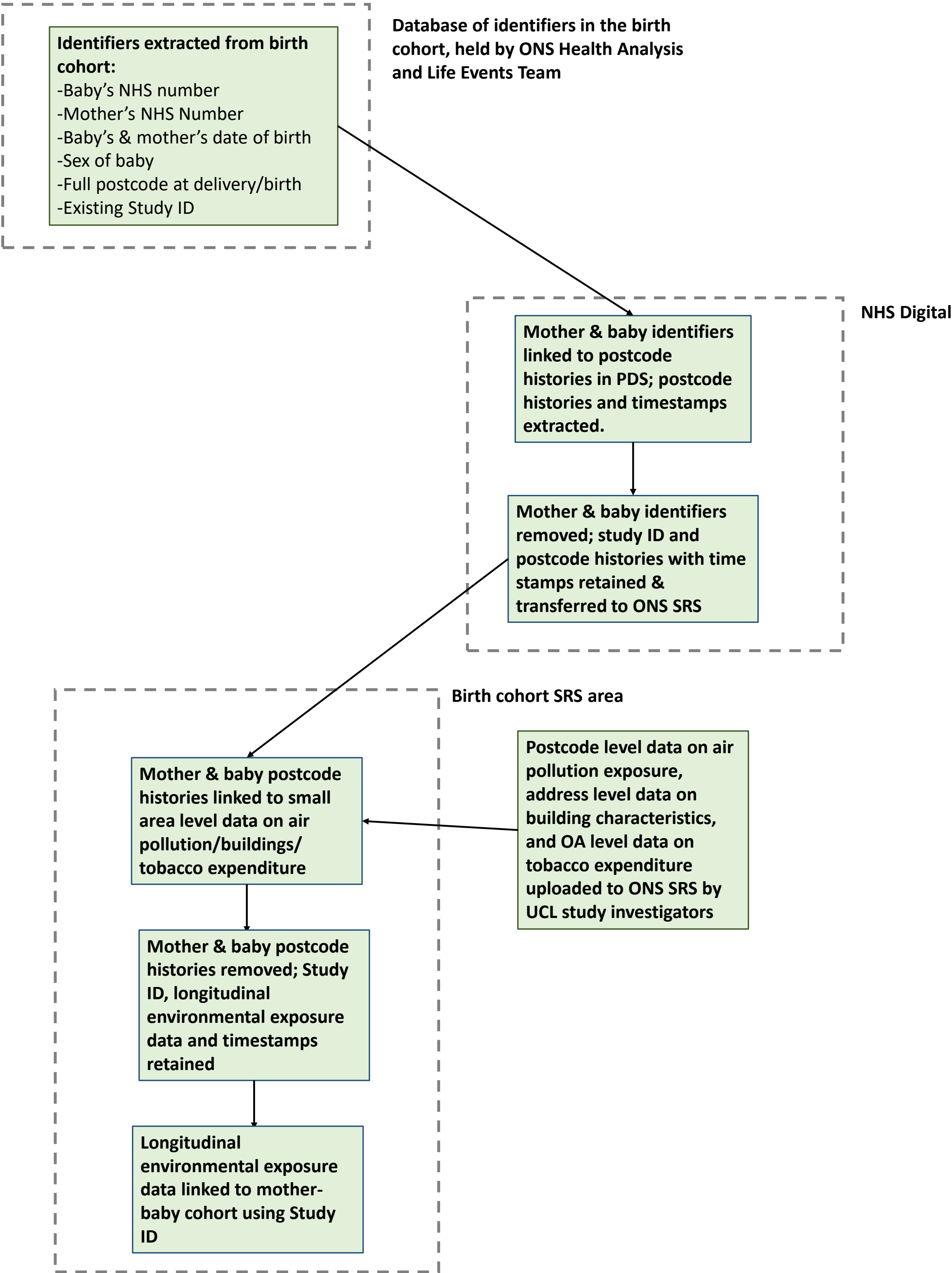

Supplement: Supplementary data [file bmjopen-2020-048038supp002.pdf]

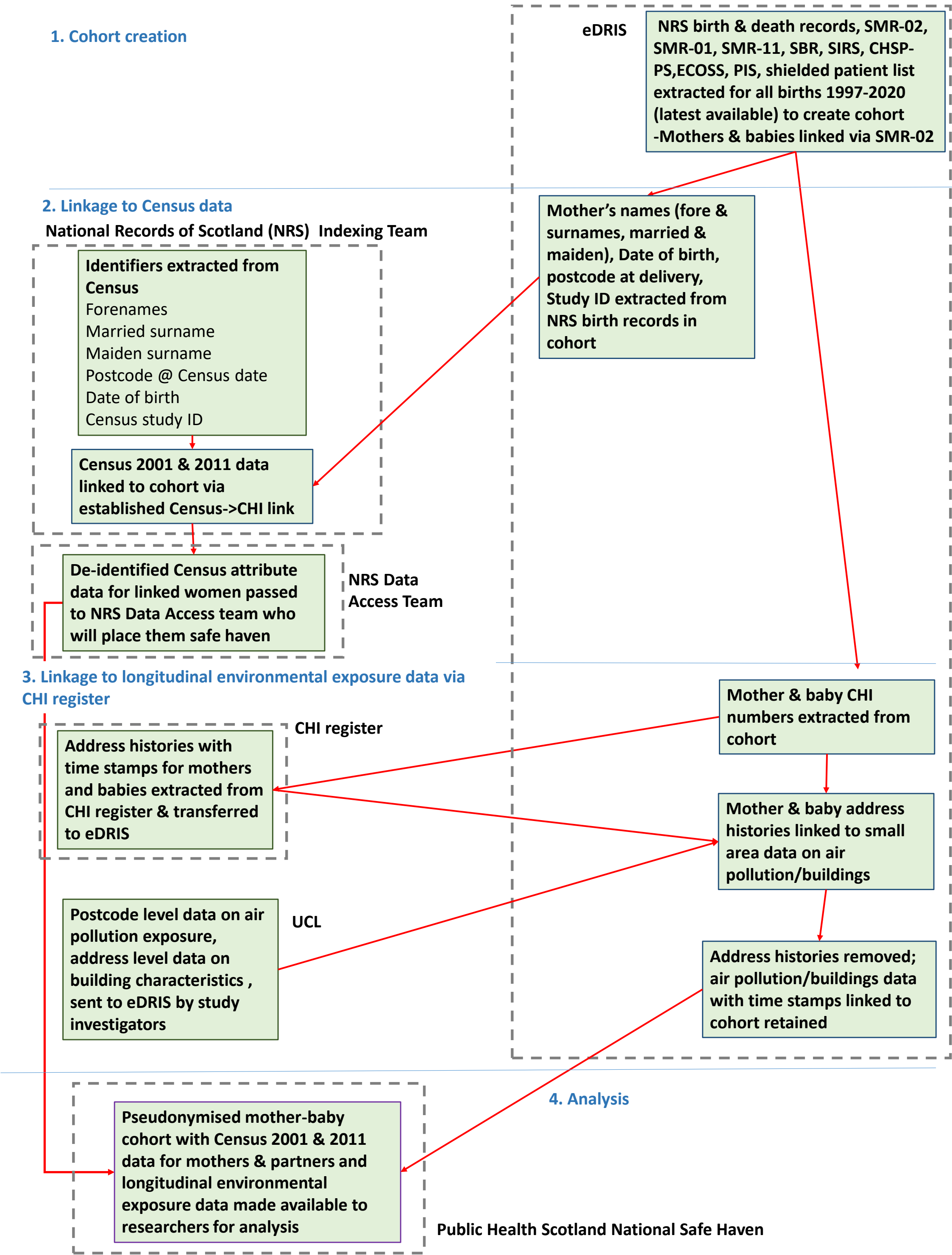

Supplement: Supplementary data [file bmjopen-2020-048038supp003.pdf]
