# Supplementary material for: Air Pollution, housing and respiratory tract Infections in Children: NatIonal birth Cohort study (PICNIC): study protocol
Source: BMJ Open. 2021 May 3;11(5):e048038. doi: 10.1136/bmjopen-2020-048038 (PMC8098990; doi:10.1136/bmjopen-2020-048038)
Supplement: Author's manuscript [file bmjopen-2020-048038.draft_revisions.pdf]

BMJ Open is committed to open peer review. As part of this commitment we make the peer review history of every article we publish publicly available.

When an article is published we post the peer reviewers' comments and the authors' responses online. We also post the versions of the paper that were used during peer review. These are the versions that the peer review comments apply to.

The versions of the paper that follow are the versions that were submitted during the peer review process. They are not the versions of record or the final published versions. They should not be cited or distributed as the published version of this manuscript.

BMJ Open is an open access journal and the full, final, typeset and author-corrected version of record of the manuscript is available on our site with no access controls, subscription charges or pay-per-view fees (http://bmjopen.bmj.com).

If you have any questions on BMJ Open's open peer review process please email info.bmjopen@bmj.com

BMJ Open

Air Pollution, housing and respiratory tract Infections in Children: NatIonal birth Cohort study (PICNIC) - study protocol

Journal:	*BMJ Open*
Manuscript ID	bmjopen-2020-048038
Article Type:	Protocol
Date Submitted by the Author:	17-Dec-2020

Complete List of Authors:	Favarato, Graziella; University College London Institute of Child Health, Population, Policy & Practice Research and Teaching Department<br>Clemens, Tom; The University of Edinburgh, School of Geosciences<br>Cunningham, Steven; University of Edinburgh, Centre for Inflammation Research<br>Dibben, Chris; The University of Edinburgh,, School of Geosciences<br>Macfarlane, Alison; City University, Dept of Midwifery<br>Milojevic, Ai; London School of Hygiene & Tropical Medicine, Dept of Social and Environmental Health Research<br>Taylor, Jonathon; Tampere University, Faculty of Built Environment<br>Wijlaars, Linda; University College London Institute of Child Health, Population, Policy and Practice Research and Teaching Department<br>Wood, Rachael; Public Health Scotland, Clinical and Public Health Intelligence Team; University of Edinburgh, Centre for Clinical Brain Sciences<br>Hardelid, Pia; University College London Institute of Child Health, Population, Policy & Practice Research and Teaching Department

Keywords:	EPIDEMIOLOGY, Public health < INFECTIOUS DISEASES, PAEDIATRICS, Epidemiology < THORACIC MEDICINE, Respiratory infections < THORACIC MEDICINE

SCHOLARONE™
Manuscripts

I, the Submitting Author has the right to grant and does grant on behalf of all authors of the Work (as defined in the below author licence), an exclusive licence and/or a non-exclusive licence for contributions from authors who are: i) UK Crown employees; ii) where BMJ has agreed a CC-BY licence shall apply, and/or iii) in accordance with the terms applicable for US Federal Government officers or employees acting as part of their official duties; on a worldwide, perpetual, irrevocable, royalty-free basis to BMJ Publishing Group Ltd ("BMJ") its licensees and where the relevant Journal is co-owned by BMJ to the co-owners of the Journal, to publish the Work in this journal and any other BMJ products and to exploit all rights, as set out in our licence.

The Submitting Author accepts and understands that any supply made under these terms is made by BMJ to the Submitting Author unless you are acting as an employee on behalf of your employer or a postgraduate student of an affiliated institution which is paying any applicable article publishing charge ("APC") for Open Access articles. Where the Submitting Author wishes to make the Work available on an Open Access basis (and intends to pay the relevant APC), the terms of reuse of such Open Access shall be governed by a Creative Commons licence – details of these licences and which Creative Commons licence will apply to this Work are set out in our licence referred to above.

Other than as permitted in any relevant BMJ Author's Self Archiving Policies, I confirm this Work has not been accepted for publication elsewhere, is not being considered for publication elsewhere and does not duplicate material already published. I confirm all authors consent to publication of this Work and authorise the granting of this licence.

For peer review only

Air Pollution, housing and respiratory tract Infections in Children: NatIonal birth Cohort study (PICNIC) - study protocol

G Favarato,[1] T Clemens,[2] S Cunningham,[3] C Dibben,[3] A Macfarlane,[4] A Milojevic,[5] J Taylor,[6] L Wijlaars,[1] R Wood[7,8] P Hardelid[1*]

[1]Population, Policy & Practice Research & Teaching Department, UCL Great Ormond Street Institute of Child Health, London, UK
[2]School of Geosciences, University of Edinburgh, Edinburgh, UK
[3]Centre for Inflammation Research, University of Edinburgh, Edinburgh, UK
[4]School of Health Sciences, City, University of London, London, UK
[5]Faculty of Public Health and Policy, London School of Hygiene and Tropical Medicine, London, UK
[6]Faculty of Built Environment, Tampere University, Tampere, Finland
[7]Clinical and Public Health Intelligence Team; Public Health Scotland, Edinburgh, UK
[8]Centre for Clinical Brain Sciences, University of Edinburgh, Edinburgh, UK

*Corresponding author. Address for correspondence: UCL Great Ormond Street Institute of Child Health, 30 Guilford Street, London WC1N 1EH, UK. Email: p.hardelid@ucl.ac.uk

For peer review only

Abstract

Introduction
Respiratory tract infections (RTIs) are the most common reason for hospital admission among children <5 years in the UK. The relative contribution of ambient air pollution exposure and adverse housing conditions to RTI admissions in young children is unclear, and has not been assessed in a UK context.

Methods and analysis
The aim of PICNIC is to quantify the extent to which in-utero, infant and childhood exposure to ambient air pollution, and adverse housing conditions are associated with the risk of RTI admissions in children less than 5 years old. We will use national administrative data birth cohorts, including data from all children born in England in 2005-2014, and in Scotland 1997-2020, created via linkage between civil registration, maternity and hospital admission datasets. We will further enhance these cohorts via linkage to Census data on housing conditions and socio-economic position, and small-area level data on ambient air pollution and building characteristics. We will use time-to-event analyses to examine the association between air pollution, housing characteristics and the risk of RTI admissions in children, calculate population attributable fractions for ambient air pollution and housing characteristics, and use causal mediation analyses to explore the mechanisms through which housing and air pollution influences the risk of infant RTI admission.

Ethics, expected impact and dissemination
To date, we have obtained approval from six ethics and information governance committees in England, and two in Scotland. Our results will inform parents, national and local government, the National Health Service and voluntary sector organisations of the relative contribution of adverse housing conditions and air pollution to RTI admissions in young children. We will publish our results in open-access journals, and present our results to the public via parent groups and social media and on the PICNIC website. Code and metadata will be published on Github.

For peer review only

Strengths and weaknesses of the study

- PICNIC will use national, administrative data birth cohorts from England and Scotland, linked to small-area level data on environmental exposures and Census data on socio-economic position to examine the association between ambient air pollution and adverse housing exposures and respiratory tract infection admissions in children less than 5 years old.
- The national birth cohorts will include all children born in the two countries during specified time periods, thus minimising selection bias and allowing analyses of even relatively uncommon environmental exposures and infection outcomes
- Air pollution and building characteristics data will be linked to maternal and child postcode histories during pregnancy and early life, thus creating longitudinal environmental exposure data at national scale
- PICNIC will include an examination of the population-based risk factors for SARS-CoV-2 infection in Scottish children
- A key weakness is that only infections requiring hospital admission will be the primary outcome, thus respiratory infections not requiring healthcare contact will not be considered.

For peer review only - http://bmjopen.bmj.com/site/about/guidelines.xhtml

Introduction

Upper and lower respiratory tract infections (RTIs), including croup, bronchiolitis and pneumonia are the most common reason for hospital admission in children aged less than five years old in the UK, with 170,000 admissions in England alone in 2017.[1] RTI admission rates peak in winter months, contributing to the 'winter crisis' in the National Health Service (NHS).[2] Severe RTI symptoms in infancy and early childhood have been linked to adverse respiratory health outcomes in later childhood, including asthma.[3] We have previously shown that 79% of annual admissions for RTIs in infants <1 year old can be attributed to respiratory syncytial virus (RSV) and other viral infections for which no vaccines are currently available.[4] Thus, alternative strategies to vaccination are required to prevent RTIs and relieve the burden on children, parents and the NHS.

Low socio-economic position (SEP) is strongly associated with the risk of RTI admissions in children, even when accounting for tobacco smoke exposure.[5 6] There are a number of well-established proximal risk factors for RTI admissions through which low SEP may act to increase the risk of infection exposure, severe symptoms, and hospital admission (Figure 1).

Of these risk factors, exposure to ambient air pollution and adverse housing conditions (including overcrowding, indoor air pollution and damp/mould) are of particular policy and research interest in the UK. The UK has been breaching European Union targets for nitrogen dioxide (NO_2) levels for over ten years,[8] and 18% of homes in England do not meet the government's Decent Homes Standard.[9] A number of studies has shown that exposure to ambient air pollution (particularly fine particulate matter ($PM_{2.5}$) and NO_2 during pregnancy and early life,[10 11] and living in overcrowded[12] or damp/mouldy housing[13] increases the risk of RTIs in children. However, their joint contribution to RTI admissions in children has not been assessed in a UK context.

Aims and objectives

The overall aim of the Air Pollution, housing and respiratory tract Infections in Children: NatIonal birth Cohort study (PICNIC) is to determine the relative contribution of ambient air pollution and adverse housing conditions to the rate of RTI-related hospital admissions in children aged less than 5 years old. Since 44% of RTI admissions in children under 5 years old occur during infancy (the first year of life),[4] we will first focus on infant admissions, then extend our analyses until four years of age inclusive. We will use administrative data birth cohorts covering all children born in England and Scotland, enhanced via linkage to data from the corresponding censuses and small area level data on ambient air pollution and building characteristics.

Our specific objectives are to:

1) Estimate the association between long-term exposure to ambient air pollution during pregnancy and infancy and the rate of RTI admissions in infants

2) Derive variables indicating adverse housing conditions (eg overcrowding, air pollution indoors, damp/mould, ventilation) and estimate the association between housing exposures during pregnancy and infancy and infant RTI admissions

3) Based on the outcomes of analyses for objectives 1) and 2), estimate the relative contribution of ambient air pollution and adverse housing conditions on infant RTI admissions

For peer review only - http://bmjopen.bmj.com/site/about/guidelines.xhtml

4) Estimate the relative contribution of ambient air pollution and adverse housing conditions exposure during pregnancy, infancy and early childhood to RTI admissions in children less than five years old.

5) Establish the linked administrative data birth cohorts as resources for maternal and child health research

Since receipt of funding we have added two further objectives to the study:

6) Examine the association between environmental, clinical and socio-economic risk factors and COVID19 disease and other respiratory viruses in children and young people (this objective has been added as a response to the COVID19 pandemic)

7) Estimate the relative contribution of ambient air pollution and adverse housing conditions on community dispensed medicines for children aged less than 5 years old

Due to data availability, objectives 6) and 7) will be addressed using data from Scotland only.

Proposed methods

Study design, study population and follow-up

PICNIC is a population-based birth cohort study. The PICNIC study population includes two national birth cohorts comprising all children born in England between 2005 and 2014 inclusive and Scotland between 1997 and 2020 inclusive. Children will be followed from the discharge date of their postnatal admission until their date of death, migration out of Scotland or England (defined using methods described in Hardelid et al[6] for Scotland and Lewis et al[2] for England), or their 5th birthday, whichever occurs first, via hospital admission and death records. The Scottish birth cohort will also include longitudinal data on community dispensed drugs, microbiology test results, vaccination and health visiting (see below).

Data sources

Birth cohorts from England and Scotland

The English birth cohort covers all 6.7 million births in England between 2005 and 2014. It is based on linkage between Office for National Statistics (ONS) live and stillbirth, and death registration, NHS birth notifications and longitudinal Hospital Episode Statistics (HES) records for mothers and babies (Figure 2). The data linkage methods used to create the cohort were developed for a project led by City, University of London.[14] Methods to clean and validate the cohort has been described elsewhere. [15-17]

The Scottish Birth Cohort: The electronic Data Research and Innovation Service (eDRIS) will link data from Scottish birth registrations to maternity records (Scottish Morbidity Records [SMR -02]), Scottish Birth Records (SBR, and its predecessor, SMR-11), death registration, and longitudinal hospital data (SMR-01) for mother and baby. Linkage of these five datasets will allow the derivation of similar risk factors to in England, but importantly, SMR-02 also includes individual-level data on maternal smoking during pregnancy. The sophisticated Scottish health data linkage infrastructure will also allow linkage to information on breastfeeding and tobacco smoke exposure at child health first visit and at 6-8 weeks of age (from the Child Health Surveillance Programme -Preschool; CHSP-PS), and routine infant vaccinations from the Scottish Immunisation and Recall System (SIRS). We have requested linked data from eDRIS on births from 1997 onwards (when the new SMR-02 record was introduced), resulting in a cohort of approximately 1.4 million births.

For peer review only - http://bmjopen.bmj.com/site/about/guidelines.xhtml

Additional linkage to health data in Scotland in response to COVID19 crisis

In response to the COVID19 pandemic, we will seek to extend the linkage of the Scottish cohort to a number of further datasets. In Scotland, the birth cohort will also be linked to public health laboratory testing data (via the Electronic Communication of Surveillance in Scotland – ECOSS[18]), and the list of COVID19 shielded patients. In the UK, people were flagged as shielded if they were extremely vulnerable to COVID19 disease. This primarily included people who were substantially immunosuppressed, including those with cystic fibrosis or solid organ transplant recipients. These linkages will allow us to address objective 6.

Further, we will extend the cohort in Scotland to include community dispensing data for antibiotics (focusing particularly on amoxicillin, phenoxymethylpenicillin or erythromycin) or asthma inhalers (beta$_2$ agonists and inhaled corticosteroids) via linkage to the Prescribing Information System)[19]. Three quarters of antibiotics prescribed to children in primary care in the UK are for respiratory tract infections.[20] Therefore, although not all dispensing of amoxicillin, phenoxymethylpenicillin or erythromycin are for RTIs, dispensing of these antibiotics will serve as a useful proxy. These further linkages will allow us to address additional objective 7.

Longitudinal address records

Linkage of the birth cohort to the Personal Demographic Service (PDS – in England) & Community Health Index (CHI) Database (in Scotland) will allow us to assign longitudinal air pollution and housing exposures to the mothers and babies in the cohort. PDS and CHI represent the most complete record of address histories for all NHS patients in England[21] and Scotland[22] respectively.

Air ambient pollution data

We will primarily use Department for Environment, Food and Rural Affairs (DEFRA) open access data for annual background concentrations of eight major air pollutants[23] These air pollution concentrations have been derived from atmospheric chemistry transport models and are mapped to a 1x1 km grid across the UK for the period 2001-2019. These data have been widely used for studying the health effects of long-term exposure to air pollution in the UK.[24 25]

To take into account smaller spatial variation in exposure from traffic-derived air pollution we will use data from Open Street Map combined with data on traffic density and modelled data provided by Cambridge Environmental Research Consultants (CERC - http://www.cerc.co.uk). CERC will provide daily resolution data for $PM_{2.5}$, PM_{10}, NO_2 and O_3 for Greater London (modelled at a 100x100m grid) and the City of Glasgow (modelled at a 50x50 m grid) for the years 2010-2014 and 2009-2014 respectively.

We will use road network data openly available from Open Street Map[26] to calculate distance from each mother/child postcode centroid to major roads (motorways and A roads). Distance estimates will be combined with open, geocoded traffic intensity data from Department for Transport[27] on annual daily flows for different vehicle types between junctions on major roads.

Housing data sources

Census: The decennial censuses for the constituent nations of the UK contain rich data on housing (e.g. tenure, accommodation type, central heating type, number of rooms and occupants) as well as data on information on self-reported health and SEP (including education, car ownership and employment). A subset of births in the English cohort between 2010 and 2012 has been linked by ONS to Census 2011 data provided by the mother, and the mother's resident partner (if any) on the

For peer review only - http://bmjopen.bmj.com/site/about/guidelines.xhtml

2011 Census date (27th March). Mothers in the Scottish cohort who gave birth between 2000 and 2002 will be linked to their 2001 Census records (which took place on the 29th April), and mothers giving birth between 2010 and 2012 will be linked to their 2011 Census records.

Energy Performance Certificates contains energy efficiency data for all buildings in England, Wales and Scotland constructed, let or sold since 2007;[28] currently approximately 11.5 million unique dwellings. The EPC databases contain directly observed information on building characteristics at dwelling level including energy efficiency rating, insulation levels and total floor area. EPC data for England are openly available online; EPC data for Scotland will be provided by the Energy Savings Trust (https://energysavingtrust.org.uk/scotland) on behalf of the Scottish Government. Apart from directly observed variables from EPC, we will use building physics models[29] with inputs from the EPC database to predict indoor environmental conditions at postcode level nationally for England and Scotland, including indicators of mould/damp and indoor air pollution exposures. The *Home Energy Efficiency Database (HEED)* is also held by the Energy Savings Trust Scotland and contains Scotland-wide information on energy efficiency measures installed with government grants since 2009, including double glazing and roof insulation.

<u>Tobacco expenditure data</u>

Unlike in the Scottish cohort, individual-level data on tobacco smoke exposure during pregnancy or early life is not available in the English cohort. We will instead use modelled data on tobacco expenditure at Output Area level (on average 125 households), from CACI Ltd (https://www.caci.co.uk). These data have been used previously in studies of the association of environmental hazards and children's health outcomes in England to adjust for tobacco smoke exposure.[30]

Data linkage

The suggested data flows and linkages for the English and Scottish cohorts are summarised in appendix figures 1-3.

English cohort (appendix figures 1-2): The linkage to set up the English birth cohort has been described previously.[14 16] ONS has linked the mothers' information recorded on ONS birth records to the 2011 Census using a method based on names, postcodes and dates of birth.[31] Mothers' Census records have been linked to those of her partner using the Census household matrix. NHS Digital will link the maternal and child identifiers from the birth cohort to the PDS using mother and child NHS numbers, dates of birth and postcode at delivery/birth. Air pollution exposures and EPC data will be aggregated at postcode level for linkage to the birth cohort via the longitudinal postcode records in PDS.

Scottish cohort (Appendix figure 3) eDRIS will use well-established deterministic and probabilistic methods to link the health datasets for PICNIC. National Records for Scotland will use a similar method to ONS to link the maternal information to 2001 and 2011 Census records using names, dates of birth and postcodes at delivery. As part of the COVID19 pandemic response, eDRIS have linked the CHI database to Unique Property Reference Numbers (UPRNs), which uniquely identify individual dwellings. In Scotland, HEED and EPC records will be therefore be linked to the birth cohort via CHI using UPRNs, rather than postcodes. Ambient air pollution exposures will be linked to the mother and baby address histories in CHI using postcodes.

<u>Outcomes of interest</u>

For peer review only - http://bmjopen.bmj.com/site/about/guidelines.xhtml

Our primary outcome will be RTI related hospital admissions. We will identify admissions for upper RTIs (including croup, tonsillitis), lower RTIs (e.g. pneumonia, bronchiolitis, viral wheeze), and procedures for chronic RTI symptoms (tonsillectomies and myringotomies) in the longitudinal hospital admission data available in the HES for English birth cohort and SMR-01 for the Scottish birth cohort. Our secondary outcomes, to be investigated using the Scottish cohort only are a) community dispensed antibiotics and b) asthma medicines, c) positive test results for SARS-CoV2 and d) positive test results for other respiratory viruses, including influenza and RSV.

We will use gastrointestinal infections (GIs) as a negative control outcome for objective 1, since we hypothesise that GIs are not associated with air pollution exposure.[32]

Exposure variables

Outdoor air pollution: We will derive air pollution exposures for PM_{10}, $PM_{2.5}$, NO_2, NO_x, O_3, SO_2, benzene and CO using the DEFRA data mapped to the mother's postcodes during pregnancy and the child's postcodes during the first five years of life. The detailed CERC data for London and Glasgow will also be mapped to mother/child postcodes and used to calculate and explore the impact of trimester-specific exposures. We will treat the air pollution variables as continuous exposures in the analyses. We will use data on distance to road and traffic density to produce a 'distance to high traffic flow' variable to be included in the analyses.

Housing conditions: From Census data, we will derive indicators of housing attributes, including number of people per bedroom (a measure of overcrowding), accommodation type (also available from EPC data), and tenure. From the building characteristics data, we will extract a number of variables, including double glazing, energy efficiency and connection to mains gas, and derive the infiltration rate (that is, an estimate of how draughty the dwelling is). From the modelled EPC data, we will use estimated indoor/outdoor air pollution ratio,[33] and probability of damp or mould. These exposures will be available at dwelling level in Scotland, and at postcode level in England.

Additional variables: A number of variables that may confound or modify the association between air pollution, housing conditions and the risk of RTIs in children will be available from the linked datasets in PICNIC (see Table 1).

For peer review only - http://bmjopen.bmj.com/site/about/guidelines.xhtml

Table 1. Potential confounding or effect modifying variables available in the linked birth cohorts

**Birth registration**	**Birth registrations linked to notifications (England)/maternity records (Scotland)**	**Longitudinal hospital admission data**	**Census 2001** and 2011†**	**Additional variables**
-Month of birth<br>-Sex of baby<br>-Parents' occupation<br>-Parents' countries of birth<br>-Birth weight<br>-Marital status<br>-Type of registration (joint/sole registration)<br>-Index of multiple deprivation[34]/Scottish Index of Multiple Deprivation[35] (small-area level [~1500 people] indicators of deprivation derived from residential postcode	-Gestational age<br>-Recorded ethnic group of baby<br>-Maternal smoking status at booking**<br>-Maternal smoking status at delivery**<br>-Apgar score at 5 minutes**<br>-Parity**	-Maternal chronic conditions<br>-Baby's chronic conditions<br>-Baby's congenital anomalies<br>-Length of stay of birth admission	-Employment status<br>-Education level<br>-Length of stay in the UK (if born abroad)<br>-Knowledge of English<br>-Self reported health<br>-Disability<br>-Car ownership	-Tobacco expenditure data (at output area-level)*<br>-Infant vaccines received (from SIRS)**<br>-Breastfeeding at 6-8 weeks (from CHSP-PS)**<br>-Ethnic group of baby (from CHSP-PS)**<br>-2nd hand tobacco smoke exposure (from CHSP-PS)**

*England only **Scotland only; note parity has been derived in England using birth registration and longitudinal HES records.[14] †For mother and (in England only) mother's partner at the Census date (if a partner is present in household)

For peer review only

Statistical analyses

For objective 1, we will fit appropriate time-to-event regression models to estimate the association between ambient air pollution exposures during pregnancy and infancy and the rate of RTI hospital admissions. We will derive in-utero air pollution exposure levels from the annual air pollution data according to the number of weeks in each trimester that were spent at each address and include these trimester-specific exposures as baseline covariates in the model. To separate the effects of in-utero air pollution exposure from air pollution exposures during infancy, we will include infant air pollution exposure as a time varying covariate in the models. We will first fit single pollutant models, adjusted for all relevant confounders (identified by drawing causal diagrams of all factors assumed to be associated with RTI admission), then only include pollutants associated with the rate of RTI admission in multi-pollutant models. We will repeat the models for road distance/traffic density exposures. For London and Glasgow, we will derive variables indicating trimester-specific exposures to NO_2, $PM_{2.5}$ and O_3 using the CERC data with finer temporal and spatial resolution, and refit the models to examine whether critical exposure windows exist during pregnancy or infancy. We will carry out sensitivity analyses by refitting the models using GIs as a negative control outcome, to examine the extent of unmeasured confounding.[36]

For peer review only - http://bmjopen.bmj.com/site/about/guidelines.xhtml

To estimate the association between the rate of RTI admissions and housing (Objective 2), we will primarily use data for the subset of children with linked maternal Census data; however longitudinal EPC and HEED data will be available for the other children in the cohort. We will examine the distribution of housing and building variables among children to examine the degree to which they cluster, and how building characteristics change over pregnancy and early childhood. We will use appropriate time-to-event regression models to model the association between exposure to adverse housing/building characteristics during pregnancy and infancy and the rate of RTI admissions in infancy. Depending of the outcome of our preliminary analyses, we will either enter each housing variable into the regression models separately, or first apply dimension reducing methods (eg. latent class models) to identify meaningful combinations of housing/building characteristics. If exposure to building characteristics change substantially during childhood (due to residential moving) we will include these as time-varying covariates in the model. HEED data will be used to examine the potential impact of changes to housing conditions due to retrofitting and the subsequent impact on the risk of RTIs in children.

To achieve Objective 3 we will fit appropriate time-to-event models examining the joint contribution of ambient air pollution and housing/building characteristics to RTI admissions in infancy, by including terms for the air pollution and housing variables found to be associated with RTI admissions in objectives 1) and 2), and air pollution: housing effect modification terms. We will calculate population attributable fractions for air pollution and housing characteristics,[37] and use causal mediation analyses[38] to explore the mechanisms through which housing and air pollution influences the risk of infant RTI admission.

We will repeat analyses for Objectives 1-3, extending the follow-up time until the child's fifth birthday for objective 4. As for objectives 1-3 we will model time to first RTI event, but also examine the time to repeated events using methods for multivariate failure time data.[39] RTIs in infancy is associated with the risk of repeated wheeze episodes in later childhood.[40] Therefore we will examine whether exposure to ambient air pollution and/or adverse housing conditions increases the frequency of recurrent wheeze admissions (or salbutamol prescribing in the Scottish cohort) among children with an RTI admission in infancy.

The English and Scottish birth cohorts linked to Census and small-area level data will be extremely rich longitudinal datasets that can be used to examine the long-term consequences of environmental exposures, housing and SEP during pregnancy and early childhood. To meet objective 5, we will develop clear governance procedures and documentation for researchers wishing to access the existing cohorts, and/or link in other datasets. All our code and metadata will be published on our Github page: https://github.com/UCL-CHIG

For objective 6, we will build on our previous work examining family and clinical risk factors for influenza[41] and respiratory syncytial virus (RSV),[6] to calculate rates of SARS-CoV-2 virus positivity and associated hospital admissions in children according to clinical, family and environmental risk factors. For this objective, we will follow children from birth until age 24 years. We will use appropriate time-to-event models to estimate the independent association between the risk factors and COVID19 infection. We will repeat these analyses for other respiratory viruses, including influenza and RSV. As part of this objective we will also explore the potential to link in additional data on SARS-CoV-2 infections among the child's household contacts, to further examine the epidemiology of SARS-CoV-2 among children.

For objective 7, we will repeat our analyses for objective 3 and 4 using rates of community dispensed antibiotics and asthma inhalers as outcome variables.

For peer review only - http://bmjopen.bmj.com/site/about/guidelines.xhtml

Patient and public involvement

We have carried out several consultations with parents during the development of this proposal:

- Consulting parents in Bradford, via the Born in Bradford Study[42], on the control of respiratory infections in children (June 2016)
- Meeting with the NIHR Great Ormond Street Biomedical Research Centre Parents' Advisory Group (March 2017)
- Consulting parents with a history of housing problems, Shelter Birmingham (June 2018)

Parent involvement has led to including housing exposures and examining the combined effect of adverse housing conditions and air pollution exposure. We will work with The Clean Air Parents Network and Shelter to set up meetings with parents who have been affected by poor housing or are concerned about the health effects of air pollution to guide analyses, interpret findings and support dissemination.

Expected study size

- For objective 1 we will use all available linked data: 6.7 million children in England (1,200, 000 RTI admissions), and 1.4 million children in Scotland (~200,000 RTI admissions).
- For objective 2)-4) (including building/housing characteristics), 1.5 million children have been linked to their mothers' 2011 Census records. In Scotland, around 300,000 children will be linked to their mother's 2001 & 2011 Census records.
- Around 384,000 children born in London 2010-2014, and 42,000 in Glasgow 2009-14 will be linked to 100x100m and 50x50m grid daily air pollution exposure data respectively.

Ethics and dissemination

<u>Ethics and research governance</u>

We have obtained, or are in the process of obtaining ethical or information governance approvals from the following committees:

England

- NHS London Queen's Square Ethics Committee (original application approved and amendment under review; reference: 18/LO/1514)
- Confidentiality Advisory group (original application approved and amendment under review, reference: 18/CAG/0159)
- Administrative Data Research Network (approval, reference PROJ-194 – note committee is now defunct)
- ONS Research Accreditation Panel (full approval, reference 2019/020)
- National Statistician's Data Ethics Advisory Committee (full approval, reference: 18(07))
- Independent Group Advising on Release of Data (NHS Digital; original application approved and amendment under review DARS-NIC-234656)

Scotland

- Public Benefit and Privacy Committee- Health and Social Care (full approval, reference 1819-0049)

For peer review only - http://bmjopen.bmj.com/site/about/guidelines.xhtml

For peer review only

- Public Benefit and Privacy Committee- Statistics (application submitted reference 1819-0049)
- University of Edinburgh School of Geosciences Ethics Committee (full approval, reference 2020-401)

Data storage and access

All linked analyses datasets will be kept on secure servers at the ONS Secure Research Service and the Public Health Scotland National Safe Haven. All outputs (tables and figures) will be disclosure checked before being exported from these secure servers. All researchers accessing data will require training in information governance and data security as specified by ONS and Public Health Scotland.

Dissemination policy

Our research findings will be relevant to a broad audience of researchers from paediatric and pulmonary medicine, environment and climate research, and epidemiology and public health. We will therefore publish our findings in high impact journals across these fields. Similarly, we will present our research at national and international conferences in a range of disciplines. Our findings will be summarised using animations with links to papers on the PICNIC study website (https://www.ucl.ac.uk/child-health/research/population-policy-and-practice-research-and-teaching-department/cenb-clinical-12), and we will work with the UCL press office to disseminate our results to a broad audience. Cohort descriptions will be published in peer-reviewed journals.

For peer review only - http://bmjopen.bmj.com/site/about/guidelines.xhtml

Funding

PICNIC is funded by the UKRI Medical Research Council, grant reference number MR/T016558/1. Research at the UCL Great Ormond Street Institute of Health Research benefits from funding from the Great Ormond Street Biomedical Research Centre (grant reference number IS-BRC-1215-20012).

Acknowledgements

We would like to thank Bianca De Stavola, also a co-investigator of PICNIC for her input into the PICNIC study, and Diane Rennie at Public Health Scotland for her help with the PBPP application.

Author contributions

The original proposal was prepared by PH as the principal investigator of the study with input from AM, AMF, JT, CD, TC and SC. GF and PH prepared a draft of this manuscript, with input from all authors.

Competing interests

None declared

For peer review only

For peer review only - http://bmjopen.bmj.com/site/about/guidelines.xhtml

REFERENCES

1. NHS Digital. Hospital Admitted Patient Care Activity, 2017-18 2018 [Available from: https://tinyurl.com/y8w8lm5y accessed 16/04/2019.
2. Lewis K, De Stavola B, Hardelid P. Is socioeconomic position associated with bronchiolitis seasonality? A cohort study. *Journal of Epidemiology and Community Health* 2020:jech-2019-213056. doi: 10.1136/jech-2019-213056
3. Wu P, Hartert TV. Evidence for a causal relationship between respiratory syncytial virus infection and asthma. *Expert Rev Anti Infect Ther* 2011;9(9):731-45. doi: 10.1586/eri.11.92
4. Reeves RM, Hardelid P, Gilbert R, et al. Estimating the burden of respiratory syncytial virus (RSV) on respiratory hospital admissions in children less than five years of age in England, 2007-2012. *Influenza Other Respir Viruses* 2017;11(2):122-29. doi: 10.1111/irv.12443
5. Reeves RM, Hardelid P, Gilbert R, et al. Epidemiology of laboratory-confirmed respiratory syncytial virus infection in young children in England, 2010-2014: the importance of birth month. *Epidemiol Infect* 2016;144(10):2049-56. doi: 10.1017/S0950268816000352
6. Hardelid P, Verfuerden M, McMenamin J, et al. The contribution of child, family and health service factors to respiratory syncytial virus (RSV) hospital admissions in the first 3 years of life: birth cohort study in Scotland, 2009 to 2015. *Euro Surveill* 2019;24(1) doi: 10.2807/1560-7917.ES.2019.24.1.1800046
7. Heikkinen T, Chonmaitree T. Importance of respiratory viruses in acute otitis media. *Clin Microbiol Rev* 2003;16(2):230-41.
8. National Audit Office. Air Quality, 2017. https://www.nao.org.uk/wp-content/uploads/2017/11/Air-quality-Summary.pdf Accessed 12/02/2018
9. Ministry of Housing Communities and Local Government. English Housing Survey Headline Report, 2018-19 2020. https://assets.publishing.service.gov.uk/government/uploads/system/uploads/attachment_data/file/860076/2018-19_EHS_Headline_Report.pdf Accessed 13/10/2020
10. MacIntyre EA, Gehring U, Molter A, et al. Air pollution and respiratory infections during early childhood: an analysis of 10 European birth cohorts within the ESCAPE Project. *Environ Health Perspect* 2014;122(1):107-13. doi: 10.1289/ehp.1306755
11. Jedrychowski WA, Perera FP, Spengler JD, et al. Intrauterine exposure to fine particulate matter as a risk factor for increased susceptibility to acute broncho-pulmonary infections in early childhood. *Int J Hyg Environ Health* 2013;216(4):395-401. doi: 10.1016/j.ijheh.2012.12.014
12. Colosia A, Masaquel A, Hall C, et al. Residential crowding and severe respiratory syncytial virus disease among infants and young children: A systematic literature review. *BMC Infect Dis* 2012;12:95-95. doi: 10.1186/1471-2334-12-95
13. Fisk WJ, Eliseeva EA, Mendell MJ. Association of residential dampness and mold with respiratory tract infections and bronchitis: a meta-analysis. *Environmental Health* 2010;9(1):72. doi: 10.1186/1476-069X-9-72
14. Macfarlane A, Dattani N, Gibson R, et al. Births and their outcomes by time, day and year: a retrospective birth cohort data linkage study. *Health Serv Deliv Res* 2019;7:18. doi: 10.3310/hsdr07180
15. Harper G. Linkage of Maternity Hospital Episode Statistics data to birth registration and notification records for births in England 2005–2014: Quality assurance of linkage of routine data for singleton and multiple births. *BMJ Open* 2018;8(3):e017898. doi: 10.1136/bmjopen-2017-017898
16. Dattani N, Macfarlane A. Linkage of Maternity Hospital Episode Statistics data to birth registration and notification records for births in England 2005-2014: methods. A population-based birth cohort study. *BMJ Open* 2018;8(2) doi: 10.1136/bmjopen-2017-017897

For peer review only

17. Coathup V, Macfarlane A, Quigley M. Linkage of maternity hospital episode statistics birth records to birth registration and notification records for births in England 2005–2006: quality assurance of linkage. *BMJ Open* 2020;10(10):e037885. doi: 10.1136/bmjopen-2020-037885
18. ISD Scotland. Infection Intelligence Platform (IIP): High Level Guide to IIP component datasets held by NHS National Services Scotland, 2014. https://www.isdscotland.org/Health-Topics/Health-and-Social-Community-Care/Infection-Intelligence-Platform/Data/June-2014-Guide-to-IIP-Data.pdf Accessed 13/10/2020
19. Alvarez-Madrazo S, McTaggart S, Nangle C, et al. Data Resource Profile: The Scottish National Prescribing Information System (PIS). *International Journal of Epidemiology* 2016;45(3):714-15f. doi: 10.1093/ije/dyw060
20. O'Brien K, Bellis TW, Kelson M, et al. Clinical predictors of antibiotic prescribing for acutely ill children in primary care: an observational study. *Br J Gen Pract* 2015;65(638):e585-92. doi: 10.3399/bjgp15X686497
21. NHS Digital. Personal Demographics Service 2019 https://tinyurl.com/y8vcaaz2 accessed 23/04/2019.
22. ISD Scotland. Community Health Index (CHI) Number 2016 http://www.ndc.scot.nhs.uk/Data-Dictionary/SMR-Datasets//Patient-Identification-and-Demographic-Information/Community-Health-Index-Number/ accessed 04/11/2016.
23. Department for Environment FaRA. Modelled background pollution data 2019 https://uk-air.defra.gov.uk/data/pcm-data accessed 16/12/2019.
24. Milojevic A, Niedzwiedz CL, Pearce J, et al. Socioeconomic and urban-rural differentials in exposure to air pollution and mortality burden in England. *Environ Health* 2017;16(1):104. doi: 10.1186/s12940-017-0314-5 [published Online First: 2017/10/08]
25. Dibben C, Clemens T. Place of work and residential exposure to ambient air pollution and birth outcomes in Scotland, using geographically fine pollution climate mapping estimates. *Environmental Research* 2015;140:535-41. doi: https://doi.org/10.1016/j.envres.2015.05.010
26. Open StreetMap Foundation. Open StreetMap 2020 https://www.openstreetmap.org/about accessed 13/10/2020.
27. Department for Transport. Road traffic statistics - bulk downloads 2019 https://roadtraffic.dft.gov.uk/downloads accessed 05/11/2019.
28. Ministry of Housing Communities and Local Government. Energy Performance of Buildings Data: England and Wales 2018 https://epc.opendatacommunities.org/ accessed 24/07/2018.
29. Symonds P, Taylor J, Chalabi Z, et al. Development of an England-wide indoor overheating and air pollution model using artificial neural networks. *Journal of Building Performance Simulation* 2016;9(6):606-19. doi: 10.1080/19401493.2016.1166265
30. Smith RB, Fecht D, Gulliver J, et al. Impact of London's road traffic air and noise pollution on birth weight: retrospective population based cohort study. *BMJ* 2017;359 doi: 10.1136/bmj.j5299
31. Office for National Statistics. ONS policy for safeguarding data whilst managing Admin Data Research Network projects, 2015. https://tinyurl.com/zae9dda Accessed 12/02/2018
32. Hinwood AL, De Klerk N, Rodriguez C, et al. The relationship between changes in daily air pollution and hospitalizations in Perth, Australia 1992–1998: A case-crossover study. *International Journal of Environmental Health Research* 2006;16(1):27-46. doi: 10.1080/09603120500397680
33. Taylor J, Shrubsole C, Symonds P, et al. Application of an indoor air pollution metamodel to a spatially-distributed housing stock. *Science of The Total Environment* 2019;667:390-99. doi: https://doi.org/10.1016/j.scitotenv.2019.02.341
34. Ministry of Housing Communities and Local Government. The English Indices of Deprivation 2019 2019. https://assets.publishing.service.gov.uk/government/uploads/system/uploads/attachment_data/file/835115/IoD2019_Statistical_Release.pdf Accessed 13/10/2020

For peer review only - http://bmjopen.bmj.com/site/about/guidelines.xhtml

35. Scottish Government. Scottish Index of Multiple Deprivation 2020 2020 [Available from: https://www.gov.scot/collections/scottish-index-of-multiple-deprivation-2020/.
36. Richardson DB, Keil AP, Tchetgen Tchetgen E, et al. Negative Control Outcomes and the Analysis of Standardized Mortality Ratios. *Epidemiology* 2015;26(5):727-32. doi: 10.1097/ede.0000000000000353
37. Sjölander A, Vansteelandt S. Doubly robust estimation of attributable fractions in survival analysis. *Stat Methods Med Res* 2017;26(2):948-69. doi: 10.1177/0962280214564003
38. VanderWeele T. Explanation in Causal Inference. New York: Oxford University Press 2015.
39. Gutierrez R. Parametric frailty and shared frailty survival models. *The Stata Journal* 2002;2(1):22-44.
40. Scheltema NM, Nibbelke EE, Pouw J, et al. Respiratory syncytial virus prevention and asthma in healthy preterm infants: a randomised controlled trial. *The Lancet Respiratory Medicine* 2018;6(4):257-64. doi: 10.1016/S2213-2600(18)30055-9
41. Hardelid P, Verfuerden M, McMenamin J, et al. Risk factors for admission to hospital with laboratory-confirmed influenza in young children: birth cohort study. *Eur Resp J* 2017;50(3) doi: 10.1183/13993003.00489-2017
42. Born in Bradford. About Born in Bradford [website] 2020 https://borninbradford.nhs.uk/ accessed 27/11/2020.

For peer review only

For peer review only - http://bmjopen.bmj.com/site/about/guidelines.xhtml

Figure legends

Figure 1 Factors which increase the risk of RTI admissions in children (adapted from Heikkinen & Chonmaitree (2003)[7]

Figure 2: Flowchart of linked data sources in PICNIC study

For peer review only

For peer review only - http://bmjopen.bmj.com/site/about/guidelines.xhtml

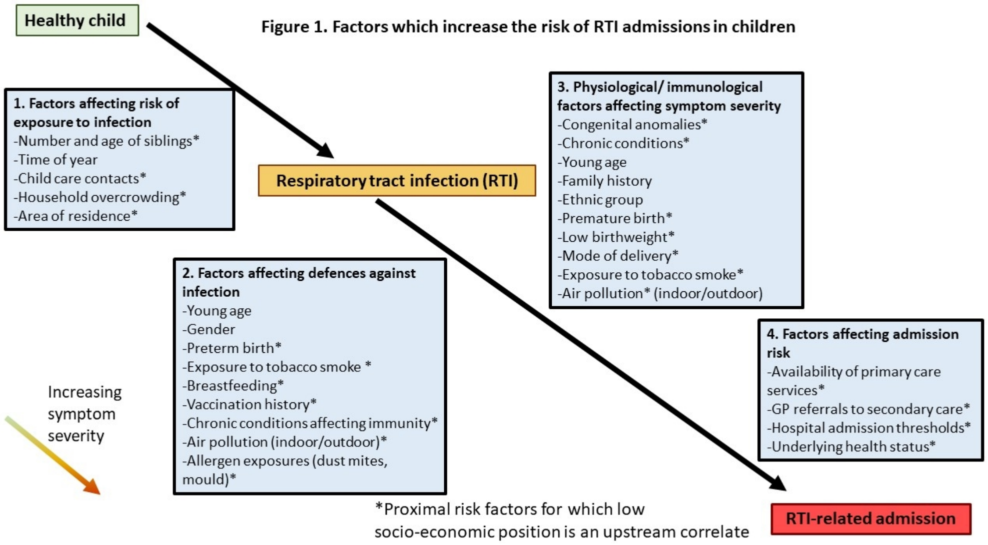

Figure 1 Factors which increase the risk of RTI admissions in children (adapted from Heikkinen & Chonmaitree (2003) [7]

338x190mm (96 x 96 DPI)

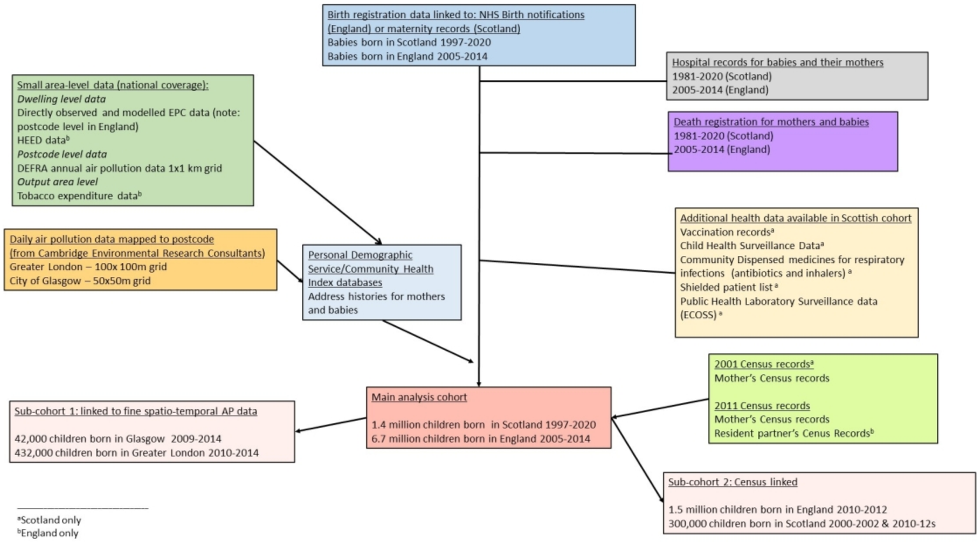

Figure 2: Flowchart of linked data sources in PICNIC study

489x275mm (96 x 96 DPI)

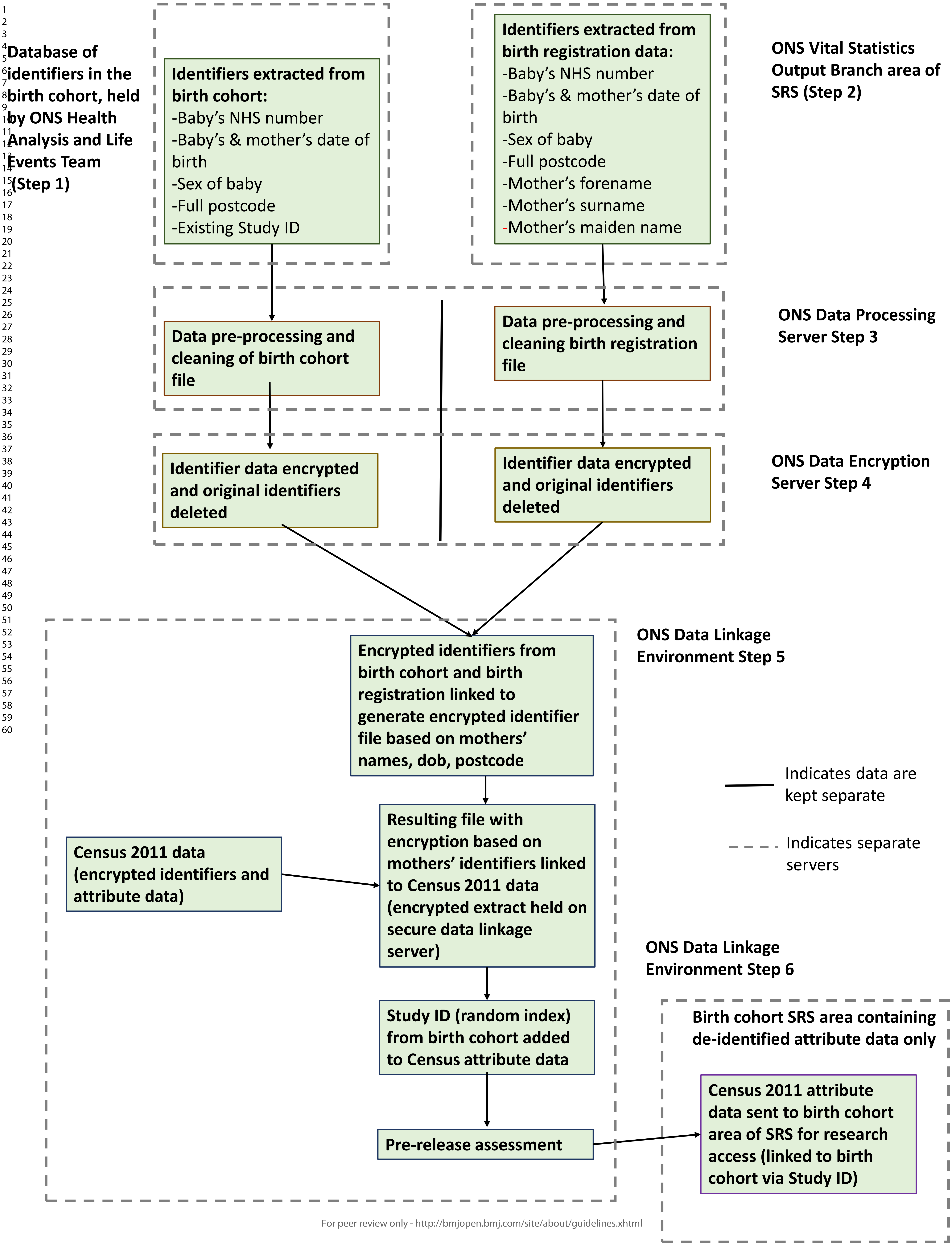
Database of identifiers in the birth cohort, held by ONS Health Analysis and Life Events Team (Step 1)
Identifiers extracted from birth cohort:
-Baby's NHS number
-Baby's & mother's date of birth
-Sex of baby
-Full postcode
-Existing Study ID
Identifiers extracted from birth registration data:
-Baby's NHS number
-Baby's & mother's date of birth
-Sex of baby
-Full postcode
-Mother's forename
-Mother's surname
-Mother's maiden name
ONS Vital Statistics Output Branch area of SRS (Step 2)
Data pre-processing and cleaning of birth cohort file
Data pre-processing and cleaning birth registration file
ONS Data Processing Server Step 3
Identifier data encrypted and original identifiers deleted
Identifier data encrypted and original identifiers deleted
ONS Data Encryption Server Step 4
Encrypted identifiers from birth cohort and birth registration linked to generate encrypted identifier file based on mothers' names, dob, postcode
ONS Data Linkage Environment Step 5
Indicates data are kept separate
Indicates separate servers
Census 2011 data (encrypted identifiers and attribute data)
Resulting file with encryption based on mothers' identifiers linked to Census 2011 data (encrypted extract held on secure data linkage server)
ONS Data Linkage Environment Step 6
Study ID (random index) from birth cohort added to Census attribute data
Birth cohort SRS area containing de-identified attribute data only
Pre-release assessment
Census 2011 attribute data sent to birth cohort area of SRS for research access (linked to birth cohort via Study ID)

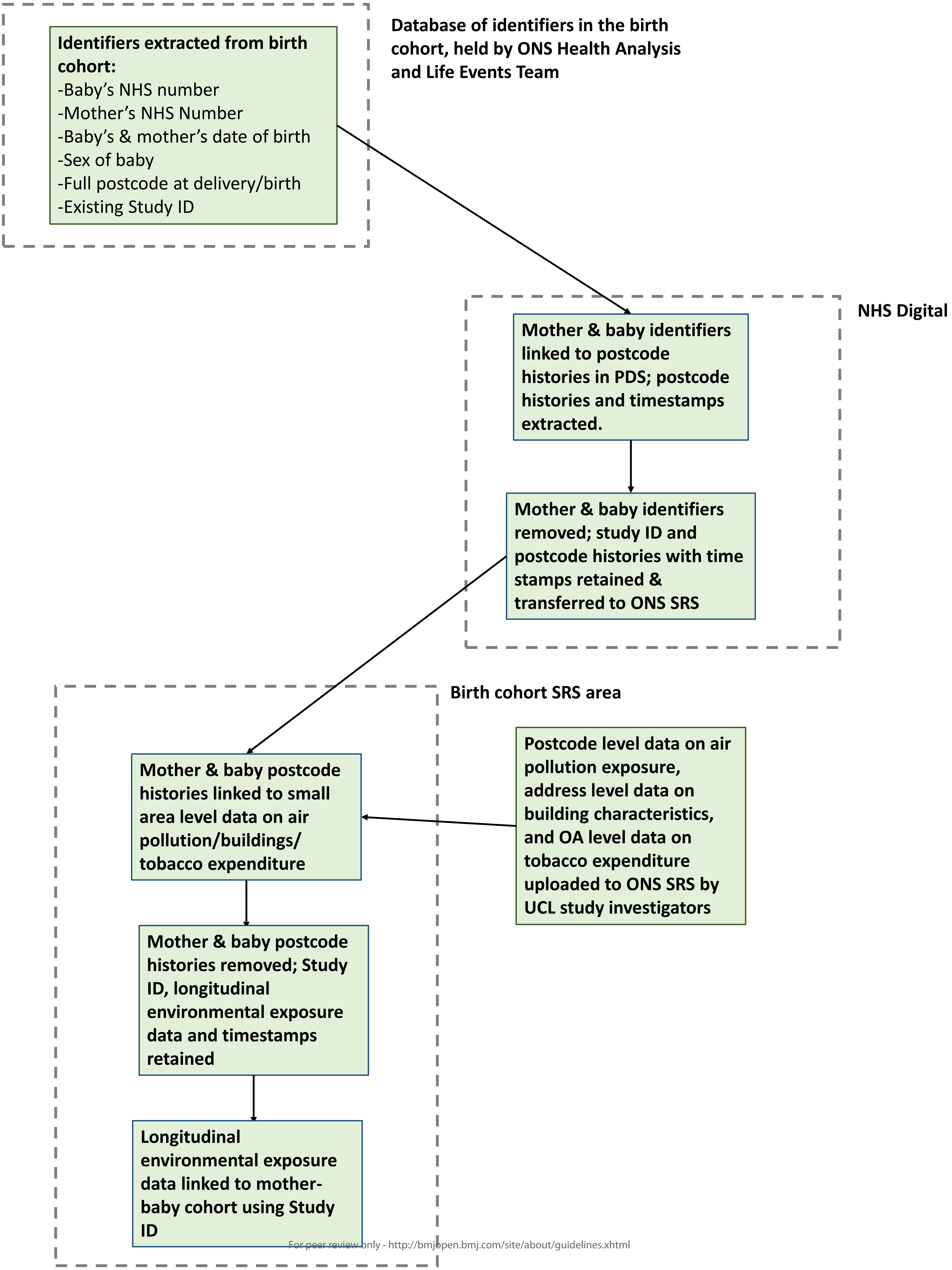
Identifiers extracted from birth cohort:
-Baby's NHS number
-Mother's NHS Number
-Baby's & mother's date of birth
-Sex of baby
-Full postcode at delivery/birth
-Existing Study ID
Database of identifiers in the birth cohort, held by ONS Health Analysis and Life Events Team
NHS Digital
Mother & baby identifiers linked to postcode histories in PDS; postcode histories and timestamps extracted.
Mother & baby identifiers removed; study ID and postcode histories with time stamps retained & transferred to ONS SRS
Birth cohort SRS area
Mother & baby postcode histories linked to small area level data on air pollution/buildings/ tobacco expenditure
Postcode level data on air pollution exposure, address level data on building characteristics, and OA level data on tobacco expenditure uploaded to ONS SRS by UCL study investigators
Mother & baby postcode histories removed; Study ID, longitudinal environmental exposure data and timestamps retained
Longitudinal environmental exposure data linked to mother-baby cohort using Study ID

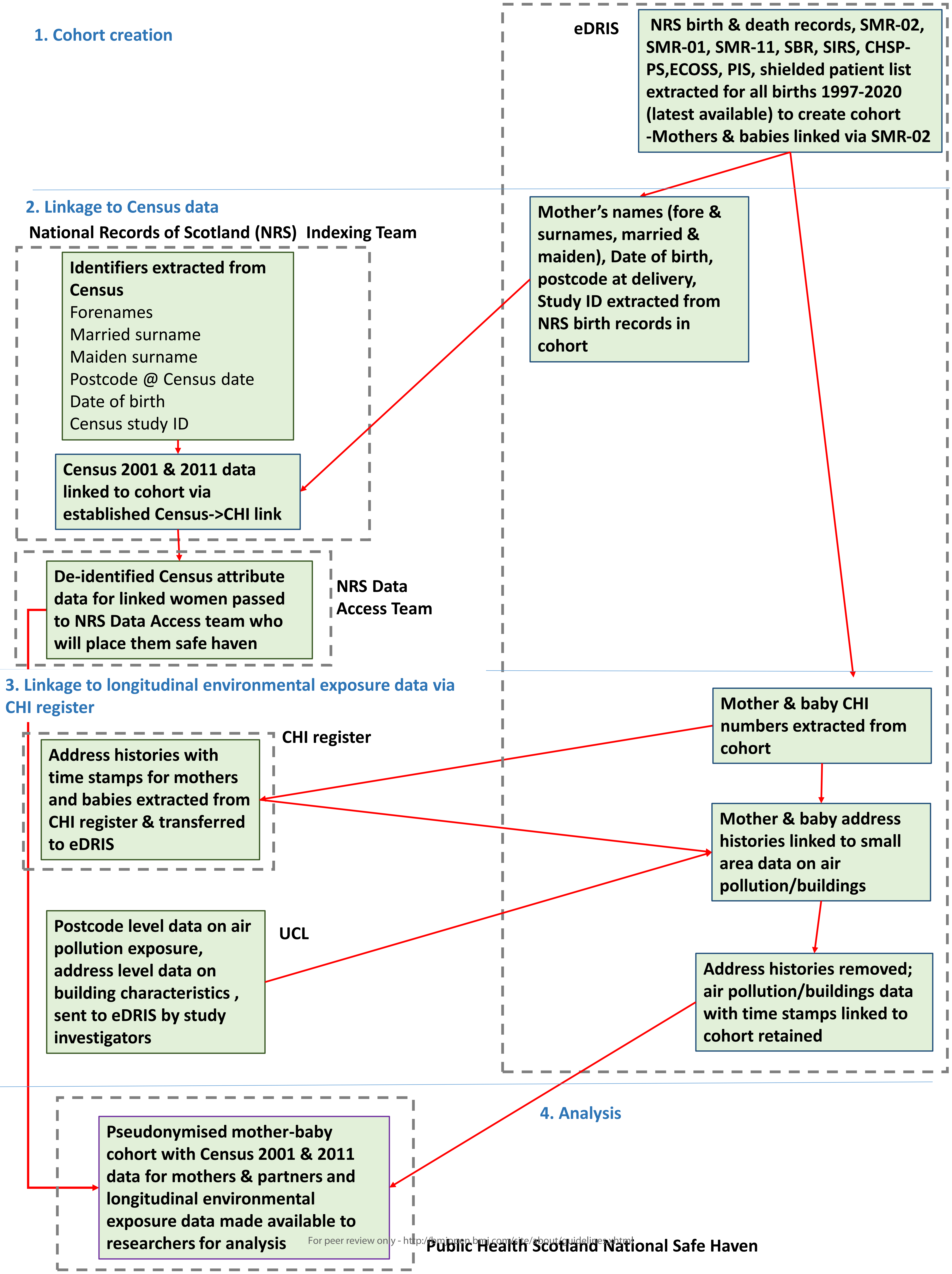
1. Cohort creation
eDRIS
NRS birth & death records, SMR-02, SMR-01, SMR-11, SBR, SIRS, CHSP-PS,ECOSS, PIS, shielded patient list extracted for all births 1997-2020 (latest available) to create cohort -Mothers & babies linked via SMR-02
2. Linkage to Census data
National Records of Scotland (NRS) Indexing Team
Identifiers extracted from Census
Forenames
Married surname
Maiden surname
Postcode @ Census date
Date of birth
Census study ID
Mother's names (fore & surnames, married & maiden), Date of birth, postcode at delivery, Study ID extracted from NRS birth records in cohort
Census 2001 & 2011 data linked to cohort via established Census->CHI link
De-identified Census attribute data for linked women passed to NRS Data Access team who will place them safe haven
NRS Data Access Team
3. Linkage to longitudinal environmental exposure data via CHI register
CHI register
Address histories with time stamps for mothers and babies extracted from CHI register & transferred to eDRIS
Mother & baby CHI numbers extracted from cohort
Mother & baby address histories linked to small area data on air pollution/buildings
Postcode level data on air pollution exposure, address level data on building characteristics , sent to eDRIS by study investigators
UCL
Address histories removed; air pollution/buildings data with time stamps linked to cohort retained
4. Analysis
Pseudonymised mother-baby cohort with Census 2001 & 2011 data for mothers & partners and longitudinal environmental exposure data made available to researchers for analysis
Public Health Scotland National Safe Haven

BMJ Open

Air Pollution, housing and respiratory tract Infections in Children: NatIonal birth Cohort study (PICNIC) - study protocol

Journal:	*BMJ Open*
Manuscript ID	bmjopen-2020-048038.R1
Article Type:	Protocol
Date Submitted by the Author:	22-Mar-2021
Complete List of Authors:	Favarato, Graziella; University College London Institute of Child Health, Population, Policy & Practice Research and Teaching Department<br>Clemens, Tom; The University of Edinburgh, School of Geosciences<br>Cunningham, Steven; University of Edinburgh, Centre for Inflammation Research<br>Dibben, Chris; The University of Edinburgh,, School of Geosciences<br>Macfarlane, Alison; City University, Dept of Midwifery<br>Milojevic, Ai; London School of Hygiene & Tropical Medicine, Dept of Social and Environmental Health Research<br>Taylor, Jonathon; Tampere University, Faculty of Built Environment<br>Wijlaars, Linda; University College London Institute of Child Health, Population, Policy and Practice Research and Teaching Department<br>Wood, Rachael; Public Health Scotland, Clinical and Public Health Intelligence Team; University of Edinburgh, Centre for Clinical Brain Sciences<br>Hardelid, Pia; University College London Institute of Child Health, Population, Policy & Practice Research and Teaching Department
**Primary Subject Heading**:	Epidemiology
Secondary Subject Heading:	Epidemiology, Infectious diseases, Paediatrics, Public health, Respiratory medicine
Keywords:	EPIDEMIOLOGY, Public health < INFECTIOUS DISEASES, PAEDIATRICS, Epidemiology < THORACIC MEDICINE, Respiratory infections < THORACIC MEDICINE

SCHOLARONE™
Manuscripts

I, the Submitting Author has the right to grant and does grant on behalf of all authors of the Work (as defined in the below author licence), an exclusive licence and/or a non-exclusive licence for contributions from authors who are: i) UK Crown employees; ii) where BMJ has agreed a CC-BY licence shall apply, and/or iii) in accordance with the terms applicable for US Federal Government officers or employees acting as part of their official duties; on a worldwide, perpetual, irrevocable, royalty-free basis to BMJ Publishing Group Ltd ("BMJ") its licensees and where the relevant Journal is co-owned by BMJ to the co-owners of the Journal, to publish the Work in this journal and any other BMJ products and to exploit all rights, as set out in our licence.

The Submitting Author accepts and understands that any supply made under these terms is made by BMJ to the Submitting Author unless you are acting as an employee on behalf of your employer or a postgraduate student of an affiliated institution which is paying any applicable article publishing charge ("APC") for Open Access articles. Where the Submitting Author wishes to make the Work available on an Open Access basis (and intends to pay the relevant APC), the terms of reuse of such Open Access shall be governed by a Creative Commons licence – details of these licences and which Creative Commons licence will apply to this Work are set out in our licence referred to above.

Other than as permitted in any relevant BMJ Author's Self Archiving Policies, I confirm this Work has not been accepted for publication elsewhere, is not being considered for publication elsewhere and does not duplicate material already published. I confirm all authors consent to publication of this Work and authorise the granting of this licence.

For peer review only

Air Pollution, housing and respiratory tract Infections in Children: NatIonal birth Cohort study (PICNIC) - study protocol

G Favarato,[1] T Clemens,[2] S Cunningham,[3] C Dibben,[3] A Macfarlane,[4] A Milojevic,[5] J Taylor,[6] L Wijlaars,[1] R Wood[7,8] P Hardelid[1*]

[1]Population, Policy & Practice Research & Teaching Department, UCL Great Ormond Street Institute of Child Health, London, UK
[2]School of Geosciences, University of Edinburgh, Edinburgh, UK
[3]Centre for Inflammation Research, University of Edinburgh, Edinburgh, UK
[4]School of Health Sciences, City, University of London, London, UK
[5]Faculty of Public Health and Policy, London School of Hygiene and Tropical Medicine, London, UK
[6]Faculty of Built Environment, Tampere University, Tampere, Finland
[7]Clinical and Public Health Intelligence Team; Public Health Scotland, Edinburgh, UK
[8]Centre for Clinical Brain Sciences, University of Edinburgh, Edinburgh, UK

*Corresponding author. Address for correspondence: UCL Great Ormond Street Institute of Child Health, 30 Guilford Street, London WC1N 1EH, UK. Email: p.hardelid@ucl.ac.uk

For peer review only

For peer review only - http://bmjopen.bmj.com/site/about/guidelines.xhtml

Abstract

Introduction

Respiratory tract infections (RTIs) are the most common reason for hospital admission among children <5 years in the UK. The relative contribution of ambient air pollution exposure and adverse housing conditions to RTI admissions in young children is unclear, and has not been assessed in a UK context.

Methods and analysis

The aim of PICNIC is to quantify the extent to which in-utero, infant and childhood exposure to ambient air pollution, and adverse housing conditions are associated with the risk of RTI admissions in children less than 5 years old. We will use national administrative data birth cohorts, including data from all children born in England in 2005-2014, and in Scotland 1997-2020, created via linkage between civil registration, maternity and hospital admission datasets. We will further enhance these cohorts via linkage to Census data on housing conditions and socio-economic position, and small-area level data on ambient air pollution and building characteristics. We will use time-to-event analyses to examine the association between air pollution, housing characteristics and the risk of RTI admissions in children, calculate population attributable fractions for ambient air pollution and housing characteristics, and use causal mediation analyses to explore the mechanisms through which housing and air pollution influences the risk of infant RTI admission.

Ethics, expected impact and dissemination

To date, we have obtained approval from six ethics and information governance committees in England, and two in Scotland. Our results will inform parents, national and local government, the National Health Service and voluntary sector organisations of the relative contribution of adverse housing conditions and air pollution to RTI admissions in young children. We will publish our results in open-access journals, and present our results to the public via parent groups and social media and on the PICNIC website. Code and metadata will be published on Github.

For peer review only

Strengths and weaknesses of the study

- PICNIC will use national, administrative data birth cohorts from England and Scotland, linked to small-area level data on environmental exposures and Census data on socio-economic position to examine the association between ambient air pollution and adverse housing exposures and respiratory tract infection admissions in children less than 5 years old.
- The national birth cohorts will include all children born in the two countries during specified time periods, thus minimising selection bias and allowing analyses of even relatively uncommon environmental exposures and infection outcomes
- Air pollution and building characteristics data will be linked to maternal and child postcode histories during pregnancy and early life, thus creating longitudinal environmental exposure data at national scale
- PICNIC will include an examination of the population-based risk factors for SARS-CoV-2 infection in Scottish children
- A key weakness is that only infections requiring hospital admission will be the primary outcome, thus respiratory infections not requiring healthcare contact will not be considered.

For peer review only

Introduction

Upper and lower respiratory tract infections (RTIs), including croup, bronchiolitis and pneumonia are the most common reason for hospital admission in children aged less than five years old in the UK, with 170,000 admissions in England alone in 2017.[1] RTI admission rates peak in winter months, contributing to the 'winter crisis' in the National Health Service (NHS).[2] Severe RTI symptoms in infancy and early childhood have been linked to adverse respiratory health outcomes in later childhood, including asthma.[3] We have previously shown that 79% of annual admissions for RTIs in infants <1 year old can be attributed to respiratory syncytial virus (RSV) and other viral infections for which no vaccines are currently available.[4] Thus, alternative strategies to vaccination are required to prevent RTIs and relieve the burden on children, parents and the NHS.

Low socio-economic position (SEP) is strongly associated with the risk of RTI admissions in children, even when accounting for tobacco smoke exposure.[5 6] There are a number of well-established proximal risk factors for RTI admissions through which low SEP may act to increase the risk of infection exposure, severe symptoms, and hospital admission (Figure 1, framework adapted from Heikkinen and Chonmaitree[7]).

Of these risk factors, exposure to ambient air pollution and adverse housing conditions (including overcrowding, indoor air pollution and damp/mould) are of particular policy and research interest in the UK. The UK has been breaching European Union targets for nitrogen dioxide (NO_2) levels for over ten years,[8] and 18% of homes in England do not meet the government's Decent Homes Standard.[9] A number of studies has shown that exposure to ambient air pollution (particularly fine particulate matter ($PM_{2.5}$) and NO_2 during pregnancy and early life,[10 11] and living in overcrowded[12] or damp/mouldy housing[13] increases the risk of RTIs in children. However, their joint contribution to RTI admissions in children has not been assessed in a UK context.

Aims and objectives

The overall aim of the Air Pollution, housing and respiratory tract Infections in Children: NatIonal birth Cohort study (PICNIC) is to determine the relative contribution of ambient air pollution and adverse housing conditions to the rate of RTI-related hospital admissions in children aged less than 5 years old. Since 44% of RTI admissions in children under 5 years old occur during infancy (the first year of life),[4] we will first focus on infant admissions, then extend our analyses until four years of age inclusive. We will use administrative data birth cohorts covering all children born in England and Scotland, enhanced via linkage to data from the corresponding censuses and small area level data on ambient air pollution and building characteristics.

Our specific objectives are to:

1) Estimate the association between long-term exposure to ambient air pollution during pregnancy and infancy and the rate of RTI admissions in infants

2) Derive variables indicating adverse housing conditions (eg overcrowding, air pollution indoors, damp/mould, ventilation) and estimate the association between housing exposures during pregnancy and infancy and infant RTI admissions

3) Based on the outcomes of analyses for objectives 1) and 2), estimate the relative contribution of ambient air pollution and adverse housing conditions on infant RTI admissions

For peer review only - http://bmjopen.bmj.com/site/about/guidelines.xhtml

4) Estimate the relative contribution of ambient air pollution and adverse housing conditions exposure during pregnancy, infancy and early childhood to RTI admissions in children less than five years old.

5) Establish the linked administrative data birth cohorts as resources for maternal and child health research

Since receipt of funding we have added two further objectives to the study:

6) Examine the association between environmental, clinical and socio-economic risk factors and COVID19 disease and other respiratory viruses in children and young people (this objective has been added as a response to the COVID19 pandemic)

7) Estimate the relative contribution of ambient air pollution and adverse housing conditions on community dispensed medicines for children aged less than 5 years old

Due to data availability, objectives 6) and 7) will be addressed using data from Scotland only.

Proposed methods

Study design, study population and follow-up

PICNIC is a population-based birth cohort study. The PICNIC study population includes two national birth cohorts comprising all children born in England between 2005 and 2014 inclusive and Scotland between 1997 and 2020 inclusive. Children will be followed from the discharge date of their postnatal admission until their date of death, migration out of Scotland or England (defined using methods described in Hardelid et al[6] for Scotland and Lewis et al[2] for England), or their 5th birthday, whichever occurs first, via hospital admission and death records. The Scottish birth cohort will also include longitudinal data on community dispensed drugs, microbiology test results, vaccination and health visiting (see below).

Data sources

Birth cohorts from England and Scotland

The English birth cohort covers all 6.7 million births in England between 2005 and 2014. It is based on linkage between Office for National Statistics (ONS) live and stillbirth, and death registration, NHS birth notifications and longitudinal Hospital Episode Statistics (HES) records for mothers and babies (Figure 2). The data linkage methods used to create the cohort were developed for a project led by City, University of London.[14] Methods to clean and validate the cohort has been described elsewhere. [15-17]

The Scottish Birth Cohort: The electronic Data Research and Innovation Service (eDRIS) will link data from Scottish birth registrations to maternity records (Scottish Morbidity Records [SMR -02]), Scottish Birth Records (SBR, and its predecessor, SMR-11), death registration, and longitudinal hospital data (SMR-01) for mother and baby. Linkage of these five datasets will allow the derivation of similar risk factors to in England, but importantly, SMR-02 also includes individual-level data on maternal smoking during pregnancy. The sophisticated Scottish health data linkage infrastructure will also allow linkage to information on breastfeeding and tobacco smoke exposure at child health first visit and at 6-8 weeks of age (from the Child Health Surveillance Programme -Preschool; CHSP-PS), and routine infant vaccinations from the Scottish Immunisation and Recall System (SIRS). We have requested linked data from eDRIS on births from 1997 onwards (when the new SMR-02 record was introduced), resulting in a cohort of approximately 1.4 million births.

For peer review only - http://bmjopen.bmj.com/site/about/guidelines.xhtml

Additional linkage to health data in Scotland in response to COVID19 crisis

In response to the COVID19 pandemic, we will seek to extend the linkage of the Scottish cohort to a number of further datasets. In Scotland, the birth cohort will also be linked to public health laboratory testing data (via the Electronic Communication of Surveillance in Scotland – ECOSS[18]), and the list of COVID19 shielded patients. In the UK, people were flagged as shielded if they were extremely vulnerable to COVID19 disease. This primarily included people who were substantially immunosuppressed, including those with cystic fibrosis or solid organ transplant recipients. These linkages will allow us to address objective 6.

Further, we will extend the cohort in Scotland to include community dispensing data for antibiotics (focusing particularly on amoxicillin, phenoxymethylpenicillin or erythromycin) or asthma inhalers (beta$_2$ agonists and inhaled corticosteroids) via linkage to the Prescribing Information System)[19]. Three quarters of antibiotics prescribed to children in primary care in the UK are for respiratory tract infections.[20] Therefore, although not all dispensing of amoxicillin, phenoxymethylpenicillin or erythromycin are for RTIs, dispensing of these antibiotics will serve as a useful proxy. These further linkages will allow us to address additional objective 7.

Longitudinal address records

Linkage of the birth cohort to the Personal Demographic Service (PDS – in England) & Community Health Index (CHI) Database (in Scotland) will allow us to assign longitudinal air pollution and housing exposures to the mothers and babies in the cohort. PDS and CHI represent the most complete record of address histories for all NHS patients in England[21] and Scotland[22] respectively.

Air ambient pollution data

We will primarily use Department for Environment, Food and Rural Affairs (DEFRA) open access data for annual background concentrations of eight major air pollutants[23] These air pollution concentrations have been derived from atmospheric chemistry transport models and are mapped to a 1x1 km grid across the UK for the period 2001-2019. These data have been widely used for studying the health effects of long-term exposure to air pollution in the UK.[24 25]

To take into account smaller spatial variation in exposure from traffic-derived air pollution we will use data from Open Street Map combined with data on traffic density and modelled data provided by Cambridge Environmental Research Consultants (CERC - http://www.cerc.co.uk). CERC will provide daily resolution data for $PM_{2.5}$, PM_{10}, NO_2 and O_3 for Greater London (modelled at a 100x100m grid) and the City of Glasgow (modelled at a 50x50 m grid) for the years 2010-2014 and 2009-2014 respectively.

We will use road network data openly available from Open Street Map[26] to calculate distance from each mother/child postcode centroid to major roads (motorways and A roads). Distance estimates will be combined with open, geocoded traffic intensity data from Department for Transport[27] on annual daily flows for different vehicle types between junctions on major roads.

Housing data sources

Census: The decennial censuses for the constituent nations of the UK contain rich data on housing (e.g. tenure, accommodation type, central heating type, number of rooms and occupants) as well as data on information on self-reported health and SEP (including education, car ownership and employment). A subset of births in the English cohort between 2010 and 2012 has been linked by ONS to Census 2011 data provided by the mother, and the mother's resident partner (if any) on the

For peer review only - http://bmjopen.bmj.com/site/about/guidelines.xhtml

2011 Census date (27th March). Mothers in the Scottish cohort who gave birth between 2000 and 2002 will be linked to their 2001 Census records (which took place on the 29th April), and mothers giving birth between 2010 and 2012 will be linked to their 2011 Census records.

Energy Performance Certificates contains energy efficiency data for all buildings in England, Wales and Scotland constructed, let or sold since 2007;[28] currently approximately 11.5 million unique dwellings. The EPC databases contain directly observed information on building characteristics at dwelling level including energy efficiency rating, insulation levels and total floor area. EPC data for England are openly available online; EPC data for Scotland will be provided by the Energy Savings Trust (https://energysavingtrust.org.uk/scotland) on behalf of the Scottish Government. Apart from directly observed variables from EPC, we will use building physics models[29] with inputs from the EPC database to predict indoor environmental conditions at postcode level nationally for England and Scotland, including indicators of mould/damp and indoor air pollution exposures. The *Home Energy Efficiency Database (HEED)* is also held by the Energy Savings Trust Scotland and contains Scotland-wide information on energy efficiency measures installed with government grants since 2009, including double glazing and roof insulation.

Tobacco expenditure data

Unlike in the Scottish cohort, individual-level data on tobacco smoke exposure during pregnancy or early life is not available in the English cohort. We will instead use modelled data on tobacco expenditure at Output Area level (on average 125 households), from CACI Ltd (https://www.caci.co.uk). These data have been used previously in studies of the association of environmental hazards and children's health outcomes in England to adjust for tobacco smoke exposure.[30]

Data linkage

The suggested data flows and linkages for the English and Scottish cohorts are summarised in appendix figures 1-3.

English cohort (appendix figures 1-2): The linkage to set up the English birth cohort has been described previously.[14 16] ONS has linked the mothers' information recorded on ONS birth records to the 2011 Census using a method based on names, postcodes and dates of birth.[31] Mothers' Census records have been linked to those of her partner using the Census household matrix. NHS Digital will link the maternal and child identifiers from the birth cohort to the PDS using mother and child NHS numbers, dates of birth and postcode at delivery/birth. Air pollution exposures and EPC data will be aggregated at postcode level for linkage to the birth cohort via the longitudinal postcode records in PDS.

Scottish cohort (Appendix figure 3) eDRIS will use well-established deterministic and probabilistic methods to link the health datasets for PICNIC. National Records for Scotland will use a similar method to ONS to link the maternal information to 2001 and 2011 Census records using names, dates of birth and postcodes at delivery. As part of the COVID19 pandemic response, eDRIS have linked the CHI database to Unique Property Reference Numbers (UPRNs), which uniquely identify individual dwellings. In Scotland, HEED and EPC records will be therefore be linked to the birth cohort via CHI using UPRNs, rather than postcodes. Ambient air pollution exposures will be linked to the mother and baby address histories in CHI using postcodes.

Outcomes of interest

For peer review only - http://bmjopen.bmj.com/site/about/guidelines.xhtml

Our primary outcome will be RTI related hospital admissions. We will identify admissions for upper RTIs (including croup, tonsillitis), lower RTIs (e.g. pneumonia, bronchiolitis, viral wheeze), and procedures for chronic RTI symptoms (tonsillectomies and myringotomies) in the longitudinal hospital admission data available in the HES for English birth cohort and SMR-01 for the Scottish birth cohort. Our secondary outcomes, to be investigated using the Scottish cohort only are a) community dispensed antibiotics and b) asthma medicines, c) positive test results for SARS-CoV2 and d) positive test results for other respiratory viruses, including influenza and RSV.

We will use gastrointestinal infections (GIs) as a negative control outcome for objective 1, since we hypothesise that GIs are not associated with air pollution exposure.[32]

Exposure variables

Outdoor air pollution: We will derive air pollution exposures for PM_{10}, $PM_{2.5}$, NO_2, NO_x, O_3, SO_2, benzene and CO using the DEFRA data mapped to the mother's postcodes during pregnancy and the child's postcodes during the first five years of life. The detailed CERC data for London and Glasgow will also be mapped to mother/child postcodes and used to calculate and explore the impact of trimester-specific exposures. We will treat the air pollution variables as continuous exposures in the analyses. We will use data on distance to road and traffic density to produce a 'distance to high traffic flow' variable to be included in the analyses.

Housing conditions: From Census data, we will derive indicators of housing attributes, including number of people per bedroom (a measure of overcrowding), accommodation type (also available from EPC data), and tenure. From the building characteristics data, we will extract a number of variables, including double glazing, energy efficiency and connection to mains gas, and derive the infiltration rate (that is, an estimate of how draughty the dwelling is). From the modelled EPC data, we will use estimated indoor/outdoor air pollution ratio,[33] and probability of damp or mould. These exposures will be available at dwelling level in Scotland, and at postcode level in England.

Additional variables: A number of variables that may confound or modify the association between air pollution, housing conditions and the risk of RTIs in children will be available from the linked datasets in PICNIC (see Table 1).

For peer review only

Table 1. Potential confounding or effect modifying variables available in the linked birth cohorts

Birth registration	Birth registrations linked to notifications (England)/maternity records (Scotland)	Longitudinal hospital admission data	Census 2001** and 2011†	Additional variables
-Month of birth<br>-Sex of baby<br>-Parents' occupation<br>-Parents' countries of birth<br>-Birth weight<br>-Marital status<br>-Type of registration (joint/sole registration)<br>-Index of multiple deprivation[34]/Scottish Index of Multiple Deprivation[35] (small-area level [~1500 people] indicators of deprivation derived from residential postcode	-Gestational age<br>-Recorded ethnic group of baby<br>-Maternal smoking status at booking**<br>-Maternal smoking status at delivery**<br>-Apgar score at 5 minutes**<br>-Parity**	-Maternal chronic conditions<br>-Baby's chronic conditions<br>-Baby's congenital anomalies<br>-Length of stay of birth admission	-Employment status<br>-Education level<br>-Length of stay in the UK (if born abroad)<br>-Knowledge of English<br>-Self reported health<br>-Disability<br>-Car ownership	-Tobacco expenditure data (at output area-level)*<br>-Infant vaccines received (from SIRS)**<br>-Breastfeeding at 6-8 weeks (from CHSP-PS)**<br>-Ethnic group of baby (from CHSP-PS)**<br>-2nd hand tobacco smoke exposure (from CHSP-PS)**

*England only **Scotland only; note parity has been derived in England using birth registration and longitudinal HES records.[14] †For mother and (in England only) mother's partner at the Census date (if a partner is present in household)

Statistical analyses

For objective 1, we will fit appropriate time-to-event regression models to estimate the association between ambient air pollution exposures during pregnancy and infancy and the rate of RTI hospital admissions. We will derive in-utero air pollution exposure levels from the annual air pollution data according to the number of weeks in each trimester that were spent at each address and include these trimester-specific exposures as baseline covariates in the model. To separate the effects of in-utero air pollution exposure from air pollution exposures during infancy, we will include infant air pollution exposure as a time varying covariate in the models. We will first fit single pollutant models, adjusted for all relevant confounders (identified by drawing causal diagrams of all factors assumed to be associated with RTI admission), then only include pollutants associated with the rate of RTI admission in multi-pollutant models. We will repeat the models for road distance/traffic density exposures. For London and Glasgow, we will derive variables indicating trimester-specific exposures to NO_2, $PM_{2.5}$ and O_3 using the CERC data with finer temporal and spatial resolution, and refit the models to examine whether critical exposure windows exist during pregnancy or infancy. We will carry out sensitivity analyses by refitting the models using GIs as a negative control outcome, to examine the extent of unmeasured confounding.[36]

For peer review only - http://bmjopen.bmj.com/site/about/guidelines.xhtml

To estimate the association between the rate of RTI admissions and housing (Objective 2), we will primarily use data for the subset of children with linked maternal Census data; however longitudinal EPC and HEED data will be available for the other children in the cohort. We will examine the distribution of housing and building variables among children to examine the degree to which they cluster, and how building characteristics change over pregnancy and early childhood. We will use appropriate time-to-event regression models to model the association between exposure to adverse housing/building characteristics during pregnancy and infancy and the rate of RTI admissions in infancy. Depending of the outcome of our preliminary analyses, we will either enter each housing variable into the regression models separately, or first apply dimension reducing methods (eg. latent class models) to identify meaningful combinations of housing/building characteristics. If exposure to building characteristics change substantially during childhood (due to residential moving) we will include these as time-varying covariates in the model. HEED data will be used to examine the potential impact of changes to housing conditions due to retrofitting and the subsequent impact on the risk of RTIs in children.

To achieve Objective 3 we will fit appropriate time-to-event models examining the joint contribution of ambient air pollution and housing/building characteristics to RTI admissions in infancy, by including terms for the air pollution and housing variables found to be associated with RTI admissions in objectives 1) and 2), and air pollution: housing effect modification terms. We will calculate population attributable fractions for air pollution and housing characteristics,[37] and use causal mediation analyses[38] to explore the mechanisms through which housing and air pollution influences the risk of infant RTI admission.

We will repeat analyses for Objectives 1-3, extending the follow-up time until the child's fifth birthday for objective 4. As for objectives 1-3 we will model time to first RTI event, but also examine the time to repeated events using methods for multivariate failure time data.[39] RTIs in infancy is associated with the risk of repeated wheeze episodes in later childhood.[40] Therefore we will examine whether exposure to ambient air pollution and/or adverse housing conditions increases the frequency of recurrent wheeze admissions (or salbutamol prescribing in the Scottish cohort) among children with an RTI admission in infancy.

The English and Scottish birth cohorts linked to Census and small-area level data will be extremely rich longitudinal datasets that can be used to examine the long-term consequences of environmental exposures, housing and SEP during pregnancy and early childhood. To meet objective 5, we will develop clear governance procedures and documentation for researchers wishing to access the existing cohorts, and/or link in other datasets. All our code and metadata will be published on our Github page: https://github.com/UCL-CHIG

For objective 6, we will build on our previous work examining family and clinical risk factors for influenza[41] and respiratory syncytial virus (RSV),[6] to calculate rates of SARS-CoV-2 virus positivity and associated hospital admissions in children according to clinical, family and environmental risk factors. For this objective, we will follow children from birth until age 24 years. We will use appropriate time-to-event models to estimate the independent association between the risk factors and COVID19 infection. We will repeat these analyses for other respiratory viruses, including influenza and RSV. As part of this objective we will also explore the potential to link in additional data on SARS-CoV-2 infections among the child's household contacts, to further examine the epidemiology of SARS-CoV-2 among children.

For objective 7, we will repeat our analyses for objective 3 and 4 using rates of community dispensed antibiotics and asthma inhalers as outcome variables.

For peer review only - http://bmjopen.bmj.com/site/about/guidelines.xhtml

Patient and public involvement

We have carried out several consultations with parents during the development of this proposal:

- Consulting parents in Bradford, via the Born in Bradford Study[42], on the control of respiratory infections in children (June 2016)
- Meeting with the NIHR Great Ormond Street Biomedical Research Centre Parents' Advisory Group (March 2017)
- Consulting parents with a history of housing problems, Shelter Birmingham (June 2018)

Parent involvement has led to including housing exposures and examining the combined effect of adverse housing conditions and air pollution exposure. We will work with The Clean Air Parents Network and Shelter to set up meetings with parents who have been affected by poor housing or are concerned about the health effects of air pollution to guide analyses, interpret findings and support dissemination.

Expected study size

- For objective 1 we will use all available linked data: 6.7 million children in England (1,200, 000 RTI admissions), and 1.4 million children in Scotland (~200,000 RTI admissions).
- For objective 2)-4) (including building/housing characteristics), 1.5 million children have been linked to their mothers' 2011 Census records. In Scotland, around 300,000 children will be linked to their mother's 2001 & 2011 Census records.
- Around 384,000 children born in London 2010-2014, and 42,000 in Glasgow 2009-14 will be linked to 100x100m and 50x50m grid daily air pollution exposure data respectively.

Ethics and dissemination

<u>Ethics and research governance</u>

We have obtained, or are in the process of obtaining ethical or information governance approvals from the following committees:

England

- NHS London Queen's Square Ethics Committee (original application approved and amendment under review; reference: 18/LO/1514)
- Confidentiality Advisory group (original application approved and amendment under review, reference: 18/CAG/0159)
- Administrative Data Research Network (approval, reference PROJ-194 – note committee is now defunct)
- ONS Research Accreditation Panel (full approval, reference 2019/020)
- National Statistician's Data Ethics Advisory Committee (full approval, reference: 18(07))
- Independent Group Advising on Release of Data (NHS Digital; original application approved and amendment under review DARS-NIC-234656)

Scotland

- Public Benefit and Privacy Committee- Health and Social Care (full approval, reference 1819-0049)

For peer review only - http://bmjopen.bmj.com/site/about/guidelines.xhtml

For peer review only

- Public Benefit and Privacy Committee- Statistics (application submitted reference 1819-0049)
- University of Edinburgh School of Geosciences Ethics Committee (full approval, reference 2020-401)

Data storage and access

All linked analyses datasets will be kept on secure servers at the ONS Secure Research Service and the Public Health Scotland National Safe Haven. All outputs (tables and figures) will be disclosure checked before being exported from these secure servers. All researchers accessing data will require training in information governance and data security as specified by ONS and Public Health Scotland.

Dissemination policy

Our research findings will be relevant to a broad audience of researchers from paediatric and pulmonary medicine, environment and climate research, and epidemiology and public health. We will therefore publish our findings in high impact journals across these fields. Similarly, we will present our research at national and international conferences in a range of disciplines. Our findings will be summarised using animations with links to papers on the PICNIC study website (https://www.ucl.ac.uk/child-health/research/population-policy-and-practice-research-and-teaching-department/cenb-clinical-12), and we will work with the UCL press office to disseminate our results to a broad audience. Cohort descriptions will be published in peer-reviewed journals.

For peer review only

For peer review only - http://bmjopen.bmj.com/site/about/guidelines.xhtml

Funding

PICNIC is funded by the UKRI Medical Research Council, grant reference number MR/T016558/1. Research at the UCL Great Ormond Street Institute of Health Research benefits from funding from the Great Ormond Street Biomedical Research Centre (grant reference number IS-BRC-1215-20012).

Acknowledgements

We would like to thank Bianca De Stavola, also a co-investigator of PICNIC for her input into the PICNIC study, and Diane Rennie at Public Health Scotland for her help with the PBPP application.

Author contributions

The original proposal was prepared by PH as the principal investigator of the study with input from AM, AMF, JT, CD, TC and SC. GF and PH prepared a draft of this manuscript, with input from LW, RW and all authors.

Competing interests

None declared

For peer review only

REFERENCES

1. NHS Digital. Hospital Admitted Patient Care Activity, 2017-18 2018 [Available from: https://tinyurl.com/y8w8lm5y accessed 16/04/2019.
2. Lewis K, De Stavola B, Hardelid P. Is socioeconomic position associated with bronchiolitis seasonality? A cohort study. *Journal of Epidemiology and Community Health* 2020:jech-2019-213056. doi: 10.1136/jech-2019-213056
3. Wu P, Hartert TV. Evidence for a causal relationship between respiratory syncytial virus infection and asthma. *Expert Rev Anti Infect Ther* 2011;9(9):731-45. doi: 10.1586/eri.11.92
4. Reeves RM, Hardelid P, Gilbert R, et al. Estimating the burden of respiratory syncytial virus (RSV) on respiratory hospital admissions in children less than five years of age in England, 2007-2012. *Influenza Other Respir Viruses* 2017;11(2):122-29. doi: 10.1111/irv.12443
5. Reeves RM, Hardelid P, Gilbert R, et al. Epidemiology of laboratory-confirmed respiratory syncytial virus infection in young children in England, 2010-2014: the importance of birth month. *Epidemiol Infect* 2016;144(10):2049-56. doi: 10.1017/S0950268816000352
6. Hardelid P, Verfuerden M, McMenamin J, et al. The contribution of child, family and health service factors to respiratory syncytial virus (RSV) hospital admissions in the first 3 years of life: birth cohort study in Scotland, 2009 to 2015. *Euro Surveill* 2019;24(1) doi: 10.2807/1560-7917.ES.2019.24.1.1800046
7. Heikkinen T, Chonmaitree T. Importance of respiratory viruses in acute otitis media. *Clin Microbiol Rev* 2003;16(2):230-41.
8. National Audit Office. Air Quality, 2017. https://www.nao.org.uk/wp-content/uploads/2017/11/Air-quality-Summary.pdf Accessed 12/02/2018
9. Ministry of Housing Communities and Local Government. English Housing Survey Headline Report, 2018-19 2020. https://assets.publishing.service.gov.uk/government/uploads/system/uploads/attachment_data/file/860076/2018-19_EHS_Headline_Report.pdf Accessed 13/10/2020
10. MacIntyre EA, Gehring U, Molter A, et al. Air pollution and respiratory infections during early childhood: an analysis of 10 European birth cohorts within the ESCAPE Project. *Environ Health Perspect* 2014;122(1):107-13. doi: 10.1289/ehp.1306755
11. Jedrychowski WA, Perera FP, Spengler JD, et al. Intrauterine exposure to fine particulate matter as a risk factor for increased susceptibility to acute broncho-pulmonary infections in early childhood. *Int J Hyg Environ Health* 2013;216(4):395-401. doi: 10.1016/j.ijheh.2012.12.014
12. Colosia A, Masaquel A, Hall C, et al. Residential crowding and severe respiratory syncytial virus disease among infants and young children: A systematic literature review. *BMC Infect Dis* 2012;12:95-95. doi: 10.1186/1471-2334-12-95
13. Fisk WJ, Eliseeva EA, Mendell MJ. Association of residential dampness and mold with respiratory tract infections and bronchitis: a meta-analysis. *Environmental Health* 2010;9(1):72. doi: 10.1186/1476-069X-9-72
14. Macfarlane A, Dattani N, Gibson R, et al. Births and their outcomes by time, day and year: a retrospective birth cohort data linkage study. *Health Serv Deliv Res* 2019;7:18. doi: 10.3310/hsdr07180
15. Harper G. Linkage of Maternity Hospital Episode Statistics data to birth registration and notification records for births in England 2005–2014: Quality assurance of linkage of routine data for singleton and multiple births. *BMJ Open* 2018;8(3):e017898. doi: 10.1136/bmjopen-2017-017898
16. Dattani N, Macfarlane A. Linkage of Maternity Hospital Episode Statistics data to birth registration and notification records for births in England 2005-2014: methods. A population-based birth cohort study. *BMJ Open* 2018;8(2) doi: 10.1136/bmjopen-2017-017897

For peer review only - http://bmjopen.bmj.com/site/about/guidelines.xhtml

17. Coathup V, Macfarlane A, Quigley M. Linkage of maternity hospital episode statistics birth records to birth registration and notification records for births in England 2005–2006: quality assurance of linkage. *BMJ Open* 2020;10(10):e037885. doi: 10.1136/bmjopen-2020-037885
18. ISD Scotland. Infection Intelligence Platform (IIP): High Level Guide to IIP component datasets held by NHS National Services Scotland, 2014. https://www.isdscotland.org/Health-Topics/Health-and-Social-Community-Care/Infection-Intelligence-Platform/Data/June-2014-Guide-to-IIP-Data.pdf Accessed 13/10/2020
19. Alvarez-Madrazo S, McTaggart S, Nangle C, et al. Data Resource Profile: The Scottish National Prescribing Information System (PIS). *International Journal of Epidemiology* 2016;45(3):714-15f. doi: 10.1093/ije/dyw060
20. O'Brien K, Bellis TW, Kelson M, et al. Clinical predictors of antibiotic prescribing for acutely ill children in primary care: an observational study. *Br J Gen Pract* 2015;65(638):e585-92. doi: 10.3399/bjgp15X686497
21. NHS Digital. Personal Demographics Service 2019 https://tinyurl.com/y8vcaaz2 accessed 23/04/2019.
22. ISD Scotland. Community Health Index (CHI) Number 2016 http://www.ndc.scot.nhs.uk/Data-Dictionary/SMR-Datasets//Patient-Identification-and-Demographic-Information/Community-Health-Index-Number/ accessed 04/11/2016.
23. Department for Environment FaRA. Modelled background pollution data 2019 https://uk-air.defra.gov.uk/data/pcm-data accessed 16/12/2019.
24. Milojevic A, Niedzwiedz CL, Pearce J, et al. Socioeconomic and urban-rural differentials in exposure to air pollution and mortality burden in England. *Environ Health* 2017;16(1):104. doi: 10.1186/s12940-017-0314-5 [published Online First: 2017/10/08]
25. Dibben C, Clemens T. Place of work and residential exposure to ambient air pollution and birth outcomes in Scotland, using geographically fine pollution climate mapping estimates. *Environmental Research* 2015;140:535-41. doi: https://doi.org/10.1016/j.envres.2015.05.010
26. Open StreetMap Foundation. Open StreetMap 2020 https://www.openstreetmap.org/about accessed 13/10/2020.
27. Department for Transport. Road traffic statistics - bulk downloads 2019 https://roadtraffic.dft.gov.uk/downloads accessed 05/11/2019.
28. Ministry of Housing Communities and Local Government. Energy Performance of Buildings Data: England and Wales 2018 https://epc.opendatacommunities.org/ accessed 24/07/2018.
29. Symonds P, Taylor J, Chalabi Z, et al. Development of an England-wide indoor overheating and air pollution model using artificial neural networks. *Journal of Building Performance Simulation* 2016;9(6):606-19. doi: 10.1080/19401493.2016.1166265
30. Smith RB, Fecht D, Gulliver J, et al. Impact of London's road traffic air and noise pollution on birth weight: retrospective population based cohort study. *BMJ* 2017;359 doi: 10.1136/bmj.j5299
31. Office for National Statistics. ONS policy for safeguarding data whilst managing Admin Data Research Network projects, 2015. https://tinyurl.com/zae9dda Accessed 12/02/2018
32. Hinwood AL, De Klerk N, Rodriguez C, et al. The relationship between changes in daily air pollution and hospitalizations in Perth, Australia 1992–1998: A case-crossover study. *International Journal of Environmental Health Research* 2006;16(1):27-46. doi: 10.1080/09603120500397680
33. Taylor J, Shrubsole C, Symonds P, et al. Application of an indoor air pollution metamodel to a spatially-distributed housing stock. *Science of The Total Environment* 2019;667:390-99. doi: https://doi.org/10.1016/j.scitotenv.2019.02.341
34. Ministry of Housing Communities and Local Government. The English Indices of Deprivation 2019 2019. https://assets.publishing.service.gov.uk/government/uploads/system/uploads/attachment_data/file/835115/IoD2019_Statistical_Release.pdf Accessed 13/10/2020

For peer review only - http://bmjopen.bmj.com/site/about/guidelines.xhtml

35. Scottish Government. Scottish Index of Multiple Deprivation 2020 2020 [Available from: https://www.gov.scot/collections/scottish-index-of-multiple-deprivation-2020/.
36. Richardson DB, Keil AP, Tchetgen Tchetgen E, et al. Negative Control Outcomes and the Analysis of Standardized Mortality Ratios. *Epidemiology* 2015;26(5):727-32. doi: 10.1097/ede.0000000000000353
37. Sjölander A, Vansteelandt S. Doubly robust estimation of attributable fractions in survival analysis. *Stat Methods Med Res* 2017;26(2):948-69. doi: 10.1177/0962280214564003
38. VanderWeele T. Explanation in Causal Inference. New York: Oxford University Press 2015.
39. Gutierrez R. Parametric frailty and shared frailty survival models. *The Stata Journal* 2002;2(1):22-44.
40. Scheltema NM, Nibbelke EE, Pouw J, et al. Respiratory syncytial virus prevention and asthma in healthy preterm infants: a randomised controlled trial. *The Lancet Respiratory Medicine* 2018;6(4):257-64. doi: 10.1016/S2213-2600(18)30055-9
41. Hardelid P, Verfuerden M, McMenamin J, et al. Risk factors for admission to hospital with laboratory-confirmed influenza in young children: birth cohort study. *Eur Resp J* 2017;50(3) doi: 10.1183/13993003.00489-2017
42. Born in Bradford. About Born in Bradford [website] 2020 https://borninbradford.nhs.uk/ accessed 27/11/2020.

For peer review only

For peer review only - http://bmjopen.bmj.com/site/about/guidelines.xhtml

Figure legends

Figure 1 Factors which increase the risk of RTI admissions in children (adapted from Heikkinen & Chonmaitree (2003)[7]

Figure 2: Flowchart of linked data sources in PICNIC study

For peer review only

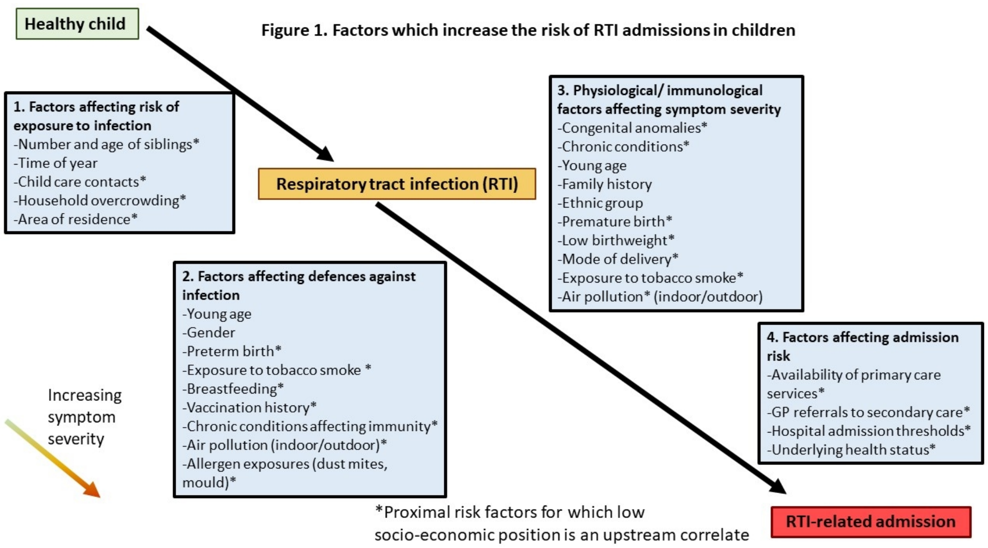

Figure 1 Factors which increase the risk of RTI admissions in children (adapted from Heikkinen & Chonmaitree (2003) [7]

338x190mm (96 x 96 DPI)

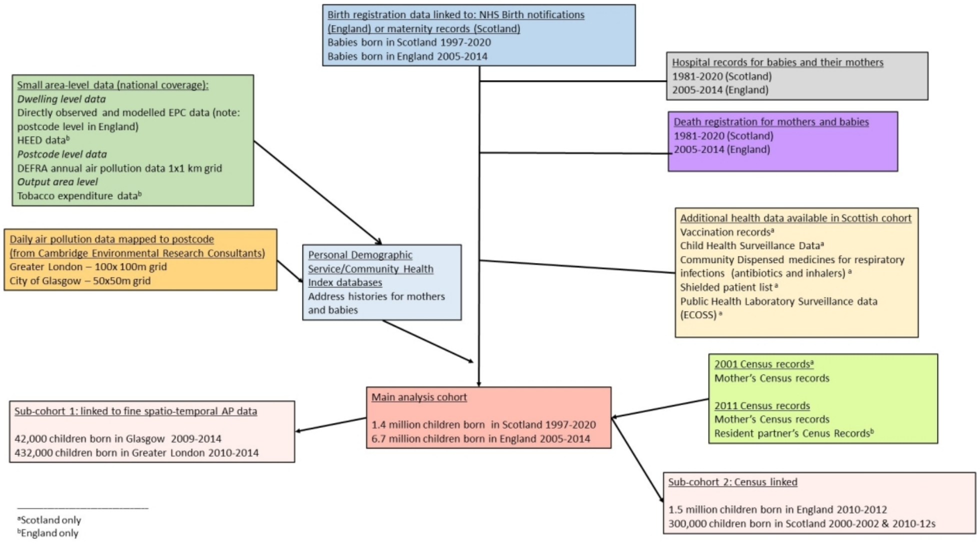

Figure 2: Flowchart of linked data sources in PICNIC study

489x275mm (96 x 96 DPI)

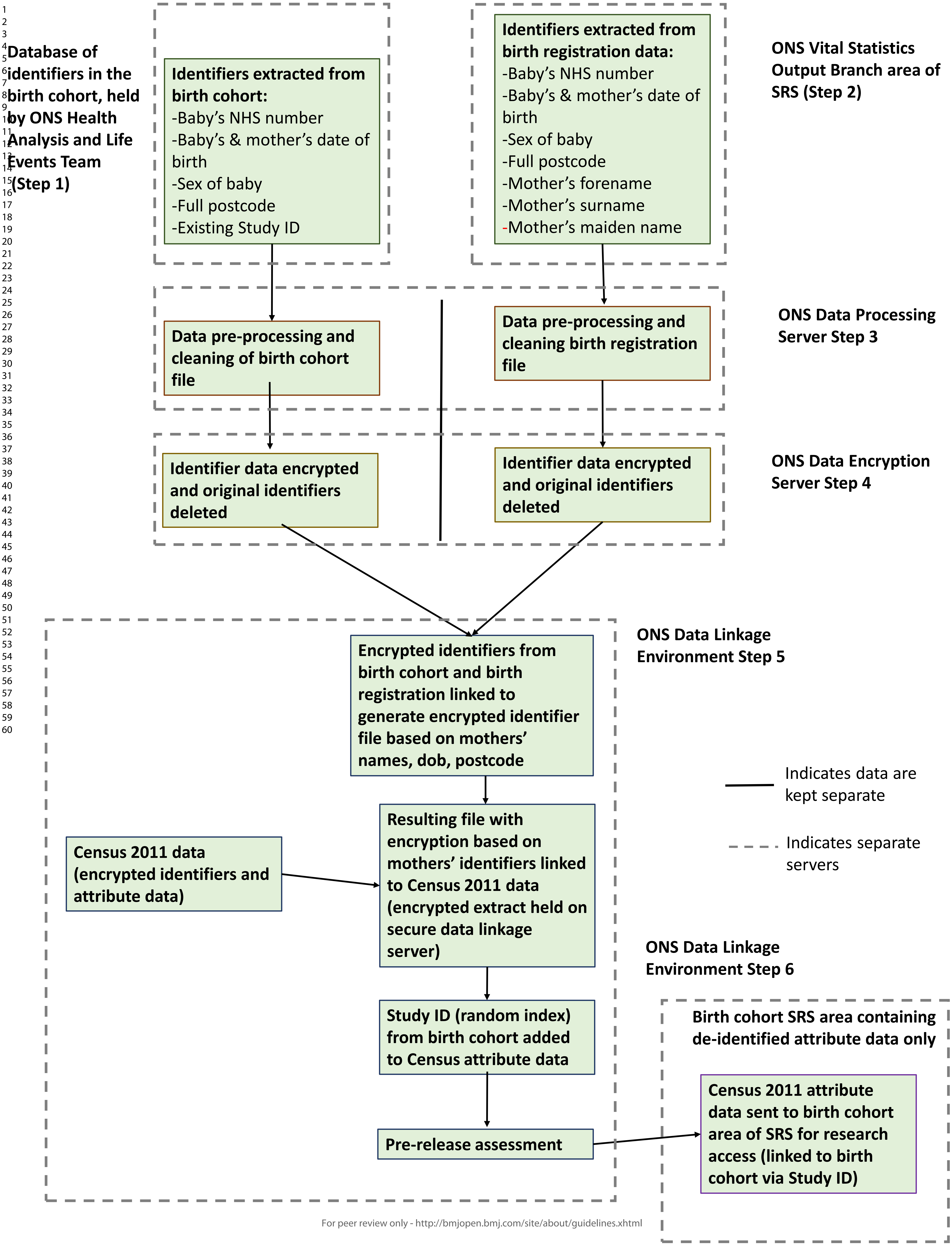
Database of identifiers in the birth cohort, held by ONS Health Analysis and Life Events Team (Step 1)
Identifiers extracted from birth cohort:
-Baby's NHS number
-Baby's & mother's date of birth
-Sex of baby
-Full postcode
-Existing Study ID
Identifiers extracted from birth registration data:
-Baby's NHS number
-Baby's & mother's date of birth
-Sex of baby
-Full postcode
-Mother's forename
-Mother's surname
-Mother's maiden name
ONS Vital Statistics Output Branch area of SRS (Step 2)
Data pre-processing and cleaning of birth cohort file
Data pre-processing and cleaning birth registration file
ONS Data Processing Server Step 3
Identifier data encrypted and original identifiers deleted
Identifier data encrypted and original identifiers deleted
ONS Data Encryption Server Step 4
ONS Data Linkage Environment Step 5
Encrypted identifiers from birth cohort and birth registration linked to generate encrypted identifier file based on mothers' names, dob, postcode
Indicates data are kept separate
Indicates separate servers
Census 2011 data (encrypted identifiers and attribute data)
Resulting file with encryption based on mothers' identifiers linked to Census 2011 data (encrypted extract held on secure data linkage server)
ONS Data Linkage Environment Step 6
Study ID (random index) from birth cohort added to Census attribute data
Birth cohort SRS area containing de-identified attribute data only
Pre-release assessment
Census 2011 attribute data sent to birth cohort area of SRS for research access (linked to birth cohort via Study ID)

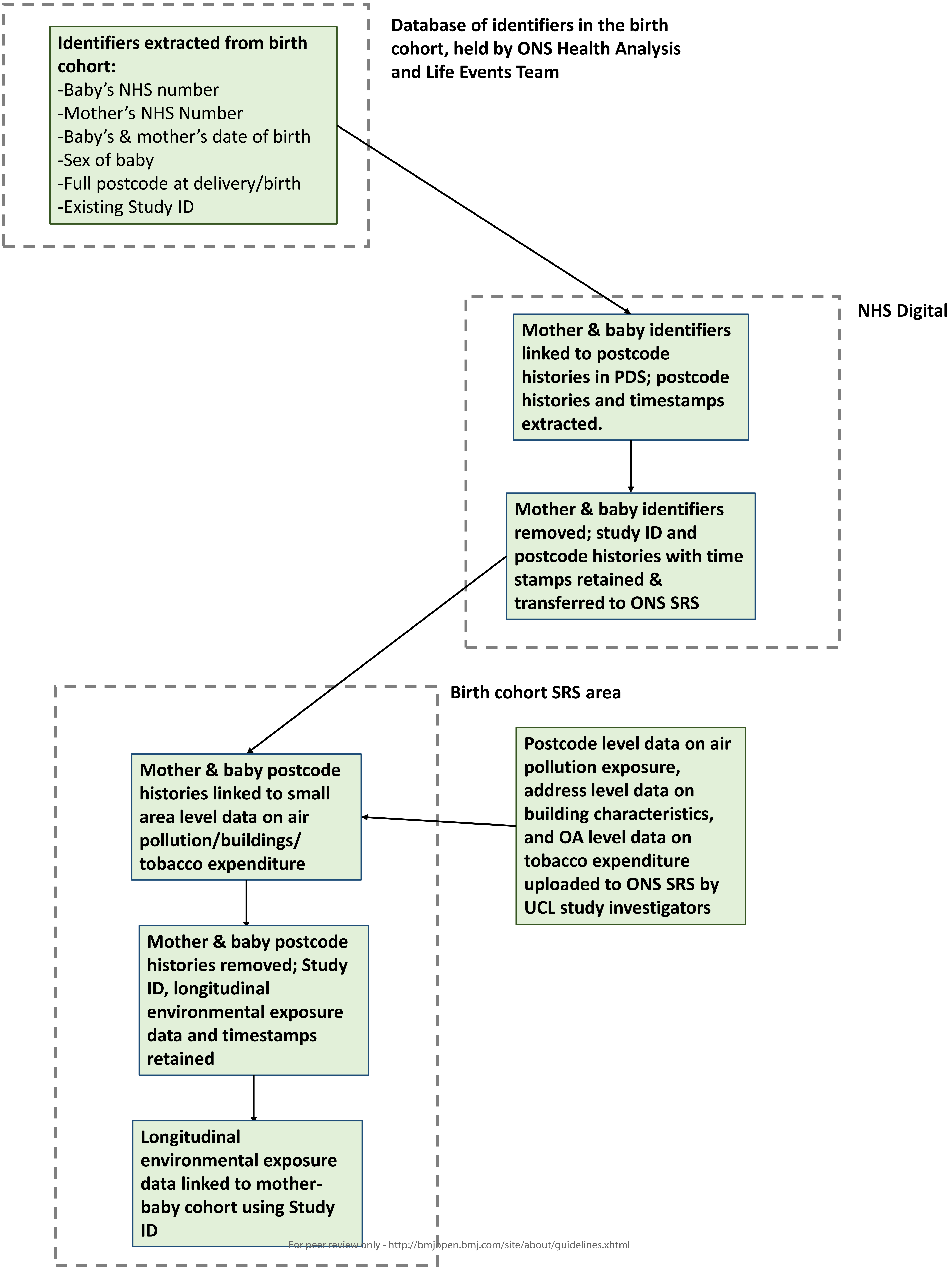
Identifiers extracted from birth cohort:
-Baby's NHS number
-Mother's NHS Number
-Baby's & mother's date of birth
-Sex of baby
-Full postcode at delivery/birth
-Existing Study ID
Database of identifiers in the birth cohort, held by ONS Health Analysis and Life Events Team
NHS Digital
Mother & baby identifiers linked to postcode histories in PDS; postcode histories and timestamps extracted.
Mother & baby identifiers removed; study ID and postcode histories with time stamps retained & transferred to ONS SRS
Birth cohort SRS area
Mother & baby postcode histories linked to small area level data on air pollution/buildings/ tobacco expenditure
Postcode level data on air pollution exposure, address level data on building characteristics, and OA level data on tobacco expenditure uploaded to ONS SRS by UCL study investigators
Mother & baby postcode histories removed; Study ID, longitudinal environmental exposure data and timestamps retained
Longitudinal environmental exposure data linked to mother-baby cohort using Study ID

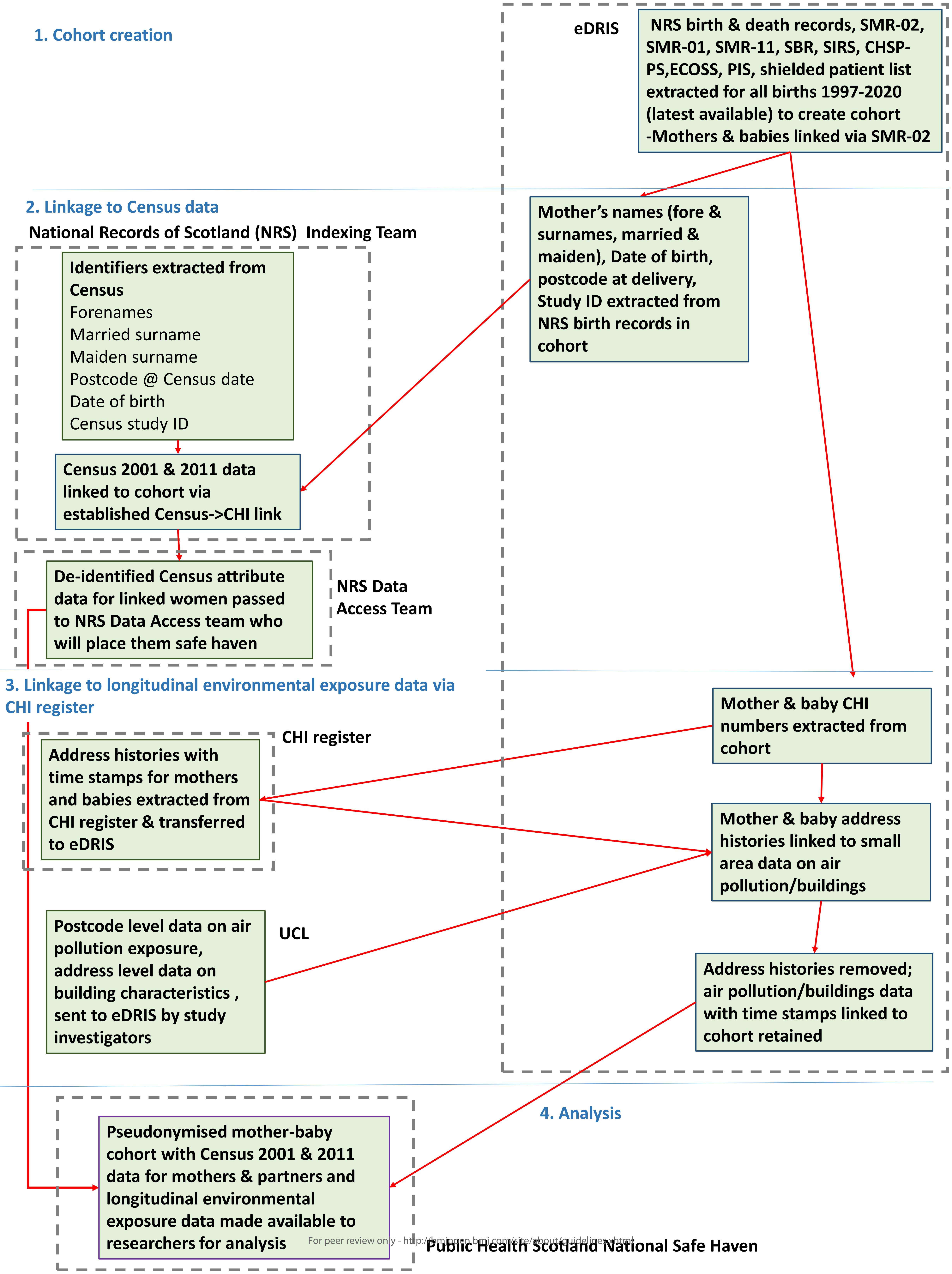
1. Cohort creation
eDRIS
NRS birth & death records, SMR-02, SMR-01, SMR-11, SBR, SIRS, CHSP-PS,ECOSS, PIS, shielded patient list extracted for all births 1997-2020 (latest available) to create cohort -Mothers & babies linked via SMR-02
2. Linkage to Census data
National Records of Scotland (NRS) Indexing Team
Identifiers extracted from Census
Forenames
Married surname
Maiden surname
Postcode @ Census date
Date of birth
Census study ID
Mother's names (fore & surnames, married & maiden), Date of birth, postcode at delivery, Study ID extracted from NRS birth records in cohort
Census 2001 & 2011 data linked to cohort via established Census->CHI link
De-identified Census attribute data for linked women passed to NRS Data Access team who will place them safe haven
NRS Data Access Team
3. Linkage to longitudinal environmental exposure data via CHI register
CHI register
Address histories with time stamps for mothers and babies extracted from CHI register & transferred to eDRIS
Mother & baby CHI numbers extracted from cohort
Mother & baby address histories linked to small area data on air pollution/buildings
Postcode level data on air pollution exposure, address level data on building characteristics , sent to eDRIS by study investigators
UCL
Address histories removed; air pollution/buildings data with time stamps linked to cohort retained
4. Analysis
Pseudonymised mother-baby cohort with Census 2001 & 2011 data for mothers & partners and longitudinal environmental exposure data made available to researchers for analysis
Public Health Scotland National Safe Haven
