# Supplementary material for: Air Pollution, housing and respiratory tract Infections in Children: NatIonal birth Cohort study (PICNIC): study protocol
Source: BMJ Open. 2021 May 3;11(5):e048038. doi: 10.1136/bmjopen-2020-048038 (PMC8098990; doi:10.1136/bmjopen-2020-048038)
Supplement: Reviewer comments [file bmjopen-2020-048038.reviewer_comments.pdf]

PEER REVIEW HISTORY

BMJ Open publishes all reviews undertaken for accepted manuscripts. Reviewers are asked to complete a checklist review form (**http://bmjopen.bmj.com/site/about/resources/checklist.pdf**) and are provided with free text boxes to elaborate on their assessment. These free text comments are reproduced below.

ARTICLE DETAILS

**TITLE (PROVISIONAL)**	Air Pollution, housing and respiratory tract Infections in Children: NatIonal birth Cohort study (PICNIC) - study protocol
**AUTHORS**	Favarato, Graziella; Clemens, Tom; Cunningham, Steven; Dibben, Chris; Macfarlane, Alison; Milojevic, Ai; Taylor, Jonathon; Wijlaars, Linda; Wood, Rachael; Hardelid, Pia

This article was not externally reviewed at BMJ Open. Protocols that have been independently assessed prior to submission to BMJ Open are usually fast-tracked to publication on the grounds that further substantial changes will not be possible. This independent assessment will usually be external, independent review for both a substantial grant award from a non-commercial or government funder and ethics approval.
